# Development of a 3D In Vitro Model of Dupuytren’s Disease as a Platform for Drug Screening

**DOI:** 10.1007/s12195-026-00885-2

**Published:** 2026-01-19

**Authors:** Jarmila Knitlova, Adam Eckhardt, Daniel Hadraba, David Vondrasek, Roman Stachon, Elena Filova, Vera Jencova, Kristyna Havlickova, Tatyana Kobets, Martin Ostadal, Lucie Bacakova

**Affiliations:** 1 Laboratory of Biomaterials and Tissue Engineering, Institute of Physiology of the Czech Academy of Sciences, Videnska 1083, 142 00 Prague 4, Czech Republic; 2 Faculty of Science, Charles University, Albertov 6, 128 00 Prague 2, Czech Republic; 3 Laboratory of Translational Metabolism, Institute of Physiology of the Czech Academy of Sciences, Videnska 1083, 14200 Prague 4, Czech Republic; 4 Laboratory of Biomathematics, Institute of Physiology of the Czech Academy of Sciences, Videnska 1083, 142 00 Prague 4, Czech Republic; 5 Department of Orthopaedics, University Hospital Bulovka, Charles University, Budinova 67/2, 180 81 Prague 8, Czech Republic; 6 Department of Chemistry, Faculty of Science, Humanities and Education, Technical University of Liberec, Studentska 1402/2, 461 17 Liberec, Czech Republic; 7 Metabolomics Service Laboratory, Institute of Physiology of the Czech Academy of Sciences, Videnska 1083, 142 00 Prague 4, Czech Republic

**Keywords:** Dupuytren’s disease, Fibrosis, 3D in vitro model, Decellularization, Myofibroblasts, Collagen type I, Minoxidil, Proteomics

## Abstract

**Background:**

Dupuytren’s disease (DD) is a common fibrotic disorder of the hand, characterized by progressive thickening and contracture of the palmar and digital fascia. Surgical excision remains the primary treatment; however, there are currently no therapies to prevent disease progression or recurrence. This study aims to develop a 3D in vitro model to test novel antifibrotic therapies. The model is based on decellularized pathological DD tissue seeded with patient-derived fibroblasts, capturing the role of both cellular and extracellular matrix components in disease progression.

**Methods:**

Fibrotic DD tissues were obtained from surgical excisions, sectioned, and decellularized. In parallel, primary fibroblasts were isolated from patient samples. The decellularized extracellular matrices (dECMs) were characterized with respect to biochemical composition, collagen structure, and mechanical properties. Fibroblasts were seeded onto the dECMs and cultured stepwise to initially promote proliferation, followed by differentiation into myofibroblasts. Secretomes of cells cultivated on the established 3D model were compared to those from conventional 2D cultivations. To evaluate the model´s relevance and effectiveness we tested the antifibrotic drug minoxidil.

**Results:**

The dECMs retained the pathological architecture and mechanical properties of native DD tissue, although individual ECM components were reduced after decellularization. Fibroblasts successfully adhered, proliferated, and repopulated the scaffold. The relevance of the 3D model was demonstrated by the presence of myofibroblasts with disease–relevant secretome. The responsiveness to the drug minoxidil was significantly more complex in the 3D model than in conventional 2D cultures.

**Conclusion:**

We demonstrated that dECM seeded with DD fibroblasts represents a relevant 3D in vitro model of Dupuytren’s disease. The model enables antifibrotic drug screening, as demonstrated by the testing of minoxidil. Our model provides a reproducible platform also suitable for the investigation of cells and ECM contributions to palmar fascial fibrosis.

**Supplementary Information:**

The online version contains supplementary material available at 10.1007/s12195-026-00885-2.

## Background

Dupuytren’s disease (DD), also known as Dupuytren’s contracture, is a progressive fibroproliferative disorder of the hand. It is manifested by the formation of thick myofibroblast-rich fibrotic nodules on the palmar fascia [1, 2]. As the disease progresses, thickened collagen-rich cords of tissue develop, extending from the palm into the fingers. The most advanced symptom is contracture of the cords, which causes a pull of the involved fingers without the ability to straighten [1–4]. Unlike fibrosis of various organs [5] DD remains underexplored, as it is not considered a life-threatening disorder, although the advanced stage is characterized by the loss of hand function and pain, which substantially affects the quality of life [3, 6]. The global prevalence of DD is 8.2% depending on ethnicity, age and gender [4, 7]. The condition typically affects middle-aged individuals, more commonly males, and its incidence increases with age. The exact aetiology of DD is not completely known, but there is an interplay of genetic factors together with other internal and external factors involved (diabetes, manual work, alcohol consumption) [1, 7].

The standard treatment is surgical fasciectomy which removes the fibrotic nodules or cords. The recurrence rate of the contracture is estimated 20–40% [8, 9]. Needle aponeurotomy and collagenase injection are other treatments that are less invasive and should ease the symptoms of DD, however both have ambiguous long term results and a higher recurrence rate than fasciectomy [8, 10]. Xiapex collagenase treatment was withdrawn from the European Union in 2020 [11]. The optimal treatment for DD would target patients in the early stages of the disease (with already palpable nodules) and prevent the cord formation which results in finger contracture. Interestingly, an intranodular injection of a TNF-α inhibitor (Adalimumab) has shown promising results in 2b clinical trials [12].

When modeling fibrosis, it is essential to recognize differentiated myofibroblasts as key effector cells producing large amounts of collagen deposited into the extracellular matrix (ECM). Shared fibrotic pathways include TGF-β, IL-6, and TNF-α signaling [13, 14], but genetic/epigenetic changes of myofibroblasts, ECM composition and tissue mechanics are disease-specific and can influence drug efficacy. In contrast to systemic organ fibrosis DD requires localized drug delivery, necessitating disease-specific models for effective therapy development.

Both in vitro and in vivo models of DD are essential for understanding the pathological mechanisms in DD and as accurate platforms for testing new, yet non-licensed, non-approved substances or treatments as well as for drug repurposing. In vivo models remain limited due to the human-specific nature of the disease. The published studies concerning in vivo DD models have been mainly based on tissue/cell transplantation into immunodeficient animals [15–18]. However, animal models, especially those based on rodents, do not adequately mimic the environment of the human organism. In addition, contemporary scientific research must incorporate ethical considerations, namely the 3Rs principles (Replacement, Reduction, and Refinement) for minimizing the use of laboratory animals [19].

Effective 3D in vitro modeling of DD requires the use of disease-specific cells and/or extracellular matrix, as these components actively interact and drive the disease progression. Studies have shown that DD-derived cells or ECM can independently activate macrophages, which then promote fibroblast migration and myofibroblast differentiation, the key processes in DD pathogenesis [20, 21]. An ex-vivo model of DD using precision-cut slices from nodular Dupuytren’s tissue was established [22, 23]. This model retains the cell and matrix complexity but has a limited incubation time, typically only two days, which does not allow sufficient time to observe appreciable treatment effects or ECM-driven cellular responses. A hydrogel-based in vitro model has also been developed to study DD. Howard et al. [24] reported increased collagen gel contraction when seeded with diseased DD cells compared to healthy patient-matched cells. Hydrogels in general have tunable mechanical properties and can be enhanced with nanomaterials or bioactive compounds to provide biochemical complexity. However, they lack the spatial organization of the native ECM. Decellularized tissues serve as 3D scaffolds for *in vitro* disease models by providing native ECM that retains the original tissue architecture and biochemical cues. These biomimetic scaffolds create a physiologically relevant microenvironment for cells, helping to better recapitulate the disease conditions *in vitro.* Their advantage compared to *ex vivo* tissue-slice models lies primarily in the higher level of experimental control, as decellularized matrices can be recellularized with defined single-cell cultures or cocultures and enable long-term observations over weeks. In addition, tissues can be stored long-term, which facilitates the inclusion of a larger number of patient samples within a single experiment [25, 26].

The aim of our study was to create a 3D in vitro model of DD based on a decelullarized ECM tissue section repopulated with DD-derived fibroblasts. To the best of our knowledge, no previous study has reported a model that incorporates both native structural cues and disease-specific cells. We intended to establish a clearly defined protocol for assembling a functional 3D model with the following key objectives:

1. To create a decellularized ECM scaffold (dECM) that preserves critical biochemical components, ultrastructure, and mechanical properties of the native DD tissue.
2. To create a 3D model of DD fibrosis by repopulating dECM with fibroblast cells isolated from Dupuytren’s tissue.
3. To demonstrate the relevance and functionality of the 3D model as a drug testing platform by application of the antifibrotic compound minoxidil in a proof-of-concept experiment.

## Materials and Methods

**Scheme 1:**
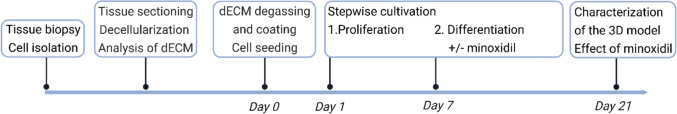
Experimental workflow

An overview of the experimental timeline is presented to provide a clear illustration of the workflow and the key steps throughout the study (Scheme 1). The details are described in the following paragraphs.

### Patient Samples

In total, samples from 53 donors were obtained during surgical fasciectomies. The overall gender ratio was: 41 male (mean age 63 ± 12 years) to 12 female (mean age 63 ± 8 years) patients. Samples were divided according to their dimensions, and the individual parts were processed separately. Portions of 53 samples were frozen in liquid nitrogen and stored at − 80 °C for decellularization. Sample portions from 8 donors (under 55 years of age) (7 males, 1 female, mean age 46 years ± 9) were used for cell isolation using the enzymatic method with slight modification of our established protocol [27]. As a modification, hyaluronidase (Sigma, M3506) at a final concentration of 330 μg/ml was added to the digestive enzyme mix. Cells from individual donors were propagated in DMEM with 10% fetal bovine serum (FBS), 25 mM HEPES buffer and gentamicin (40 μg/ml), and characterized as fibroblast cells. Flow cytometry revealed positivity of CD90 (Thy1, positivity > 98%). All cells were dim positive (without pronounced stress fibers) for myofibroblastic markers alpha smooth muscle actin (α-SMA) and intracellular ED-A fibronectin, which indicates the pre-activated protomyofibroblast cell phenotype [28]. Cells up to 4th passage were used for experiments.

The research was carried out under the Declaration of Helsinki and with the approval of the ethics committees of the participating institutions. All patients provided their written informed consent to participate.

### Decellularization

Samples were sectioned at 300 µm using a cryostat microtome (Leica CM1950). The cutting direction was parallel to the axis of tissue flexion and contraction. Sections from each sample were divided equally into 2 groups: control-unprocessed (referred to as native) and decelullarized (referred to as dECM). All samples were decontaminated overnight at 4 °C in Base 128 solution (Alchimia, BAS 006). The samples for decellularization were exposed to 0.5% w/v sodium dodecyl sulfate (SDS) in Tris buffer, pH 8, containing 5 mM EDTA, 2% antibiotic-antimycotic solution and protease inhibitors aprotinin (10 µg/ml) and leupeptin (2 mM) (all from Sigma Aldrich), for 2 h. After washing 3 times for 30 min with PBS, the samples were incubated with DNAse I (Sigma Aldrich, D4263) in Tris buffer (pH 6.8) with 25 mM MgCl_2_ and 1 mM CaCl_2_ for 30 min. After washing twice with PBS for 30 min, the final PBS washing was performed overnight at 4 °C. All incubations were performed with mild rocking and under sterile conditions.

### DNA Content

Quant-iT^TM^ Pico Green assay (Invitrogen) was used to measure the DNA content in native and decellularized samples. Briefly, the samples (n = 6) were incubated in 200 µl of proteinase K solution (final concentration 0.4 mg/ml, Thermo Fisher Scientific) in Tris-EDTA (TE) buffer at 55 °C, overnight. 200 µl of 1x TE buffer + 0.2% Triton was added to each sample and vortexed for 15 min. Samples were centrifuged at 4000 g for 5 min. The DNA content was measured in aliquots of supernatant according to the manufacturer’s protocol. The efficiency of decellularization was confirmed by imaging of nuclei labelled with DNA dye Draq5^TM^ (5 µM, Abcam, ab108410) with simultaneous second harmonic generation (SHG) imaging of type I collagen fibres (see the light microscopy section).

### Sircol Assay

The concentration of pepsin/acetic acid soluble and insoluble (crosslinked) collagen was measured in native and decellularized tissue slices (n = 6) using Sircol assay (S1000, Biocolor). The soluble fraction was extracted by overnight acidic pepsin digestion (0.1 mg/ml in 0.5 M acetic acid; pepsin EC 3.4.23.1., Sigma-Aldrich, 9001-75-6), 100 µl of pepsin per 10 mg of tissue. This digestion was repeated twice. After spinning at 3000 g for 10 min, the supernatant was harvested, and the pellet was digested for 2.5 h at 65 °C (50 µl of fragmentation reagent per 1 mg of tissue). Both supernatants were further processed according to the manufacturer’s protocol.

### Enzyme-Linked Immunosorbent Assay (ELISA)

Collagen type III and fibronectin content in native and decellularized samples (n = 7) were measured using Human Collagen III Elisa kit (LS Bio, LS-F5217) and Human Fibronectin SimpleStep ELISA kit (Abcam, ab219046), respectively. Lyophilized tissues were minced on dry ice in a tissue homogenizer (Precellys, Bertin Technologies) using ceramic beads (Qiagen, 13113-325), and proteins were released into the extraction buffer during a 30 min incubation on ice. After centrifuging (15000 g, 15 min, 4 °C), supernatants were collected for analysis. We continued the protocols according to the manufacturer’s instructions.

### Blyscan Assay

The concentration of sulphated proteoglycans and glycosaminoglycans (sGAGs) in native and decellularized samples (n = 6) was measured using Blyscan assay kit (Biocolor). Weighed lyophilized samples were subjected to papain digestion (Sigma Aldrich, P3125) in an extraction reagent prepared according to the manufacturer’s protocol. Overnight digestion at 65 °C was followed by centrifuging at 15000 g for 10 min. Supernatants were collected for measurement according to the assay protocol.

### Light Microscopy

The second-harmonic generation (SHG) signal of collagen fibers together with 2-photon excited DNA dye (Draq5^TM^, 5 mM, Abcam, ab108410) were visualized under the Bruker Ultima microscope (Bruker Corporation, Billerica, MA, USA) with 25x objective (NA 1.1, WD 2 mm, Nikon). Both signals were acquired simultaneously using two laser outputs of Chameleon Discovery TPC pulsed laser (Coherent, Santa Clara, CA, USA). The tunable laser was tuned to 810 nm for the SHG signal. The fixed laser pulses at 1040 nm to excite Draq5^TM^. The light was detected in a backscattered non-descanned direction passing a 680 nm short-pass filter. An SHG signal that is characteristic for type I collagen fibers was detected behind a bandpass filter 405/10 nm by a GaAsP detector (H11706-40P, Hamamatsu, Japan). The DNA Draq5^TM^ emission was detected behind a bandpass filter 650/50 nm by a GaAsP detector (H10770-40P, Hamamatsu, Japan). Images were merged in ImageJ FIJI software (v.1.54 k) [29].

To determine the predominant orientation of the collagen fibers as well as the fiber orientation regularity in the samples (n = 12), polarized SHG microscopy (pSHG) images were acquired using Bruker Ultima microscope with a customized half-wave plate. A linear polarized incident light beam of 810 nm was rotated at 0° - parallel to the x-axis of the image, 60° and 120° for each z-stack plane. To visualize the fiber orientation, a pixel-wise maximum intensity classification was performed. Each pixel was assigned a discrete Hue corresponding to the polarization angle exhibiting the highest intensity: 0° (Red), 60° (Green), or 120° (Blue). To preserve textural information, the Value (brightness) was defined by the maximum intensity, while the Saturation was encoded as the mean intensity across the three polarization angles. Finally, the HSV maps were converted to RGB space to produce composite images highlighting the predominant fiber orientations. The final image was converted to frequency domain and the image central moments were used for establishing the eccentricity that express the strength of fiber orientation uniformity (the value between 0 and 1, where the value 0 indicates strongly isotropic *orientation*, which is typically observed for scattered or broken fibers and levels close to 1 indicates a very strong anisotropic line-like structure in the tissue, with fibers aligned in one direction [30].

### Nanoindentation Mechanical Test

The decellularized samples and their native control samples (n = 5) were immobilized on a Petri dish using Cell-Tak™ glue (Corning®, CLS354241) at a final concentration of 3.5 µg/cm^2^ according to the manufacturer’s protocol. Then, the samples were covered with PBS buffer to prevent sample drying during the experiment and placed on the Hysitron Nanoindenter machine (Bruker, USA). The samples were probed with a spherical tip of 200.34 µm radius (Synton MDP, Switzerland). The tip was manually approached to the sample surface until reaching a setpoint force of 20 µN. The indentation was performed with a loading velocity of 1 µm/s for 16 s, followed by a 200 s relaxation phase. The same loading was repeated three more times at the same indentation site. Each sample was probed at three to four different locations in a 200 × 200 µm grid. The Young’s modulus was established as extracting the extrapolated load at infinite time *F*_*∞*_ in a multi-exponential relaxation model according to the Eq. 1

1 $${F}_{\left(t\right)}= {F}_{\infty}+\sum_{i=1}^{N}{F}_{i}exp\left(-\frac{t-{t}_{0}}{{\tau}_{i}}\right)$$

and putting the load value *F*_*∞*_ into the Hertz theory for a sphere indenter to calculate Elastic moduli (*E*) according to Eq. 2 were *ν* is a Poisson’s ratio, *R* is the radius of the sphere tip and *δ* is the total indentation depth [31].

2 $${F}_{\infty }=\frac{4E}{3(1- {\nu }^{2})}\sqrt{R}{\delta }^{3/2}$$

### Nanofiber Membrane Preparation

Polycaprolactone membrane (PCL) was prepared by DC electrospinning technique using a Nanospider^TM^ maschine NS 1WS500U (Elmarco, Liberec, Czech Republic). The polymer solution contained 10% of PCL (Mw: 80,000 g/mol, Sigma Aldrich) in a chloroform/ethanol solvent system (ratio 8:2) and electrospinning was performed as described previously [32]. The membrane characterization is provided in Additional file 1.

### Recellularization

Decelullarized samples were placed into cell non-adherent plates (Falcon 24-well plates, Corning, 351147), secured with a glass cylinder, degassed in a vacuum dryer and coated with human laminin (1 µg/cm^2^, Sigma Aldrich, L4544), 2 h at 37 °C. After the second degassing, cultured DD fibroblasts were seeded on top of dECM scaffolds (approximately 1.8 × 0.8 mm) at a density of 1 × x10^5^ cells/ well in 1.5 ml of DMEM medium (Gibco, 52100-021) containing 25 mM HEPES and gentamicin (40 μg/ml), supplemented with 10% FBS. In parallel, cells were seeded on standard polystyrene 24-well plates (referred to as 2D PS) or on polycaprolactone electrospun membranes coated with laminin (referred to as 2D PCL) at a density of 0.3 × 10^5^  cells/well. For all samples, 24 h after seeding, the culture medium was replaced with the DMEM medium supplemented with 5% of platelet lysate (Bioinova, Prague, Czech Republic) and 50 μg/ml ascorbic acid-2-phosphate (Sigma-Aldrich) (i.e., proliferation medium). After 1 week, the medium was changed to differentiation medium consisting of DMEM + 2% FBS, TGF-β1 active peptide (2.5 ng/mL, Abcam, ab50036), 50 μg/mL ascorbic acid-2-phosphate, and with/without the addition of minoxidil (Sigma Aldrich, M4145), an inhibitor of collagen crosslinking. Stock solutions of the minoxidil were prepared in 96% ethanol and stored at − 20 °C; the working solutions were diluted in differentiation culture medium to a final concentration of 0.5 mM. The total cultivation period was 3 weeks with medium changes every 3–4 days.

### Immunofluorescence Staining of Cell Laden dECM

Cell-laden dECMs were fixed with 4% paraformaldehyde for 15 min, permeabilized with 0.1% Triton and incubated for 20 min in 1% BSA in PBS for blocking. Samples were incubated with the following primary antibodies; rabbit anti-collagen I (CosmoBio, LSL-LB-1197, 1:400), mouse anti-α-SMA (Sigma-Aldrich, clone 1A4, A2547, 1:400), or mouse anti-ED-A fibronectin (Abcam, ab6328, 1:500), overnight at 4 °C with agitation. After washing with PBS, secondary antibodies, i.e., goat anti-rabbit Alexa 488 (Thermo Fisher Scientific, A11070, 1:800), goat anti-mouse Alexa 488 (Thermo Fisher Scientific, A11003, 1:800), or Alexa 633, respectively, (Thermo Fisher Scientific, A21053, 1:800) were applied, together with Hoechst 33258 (5 μg/ml) for 1 h. Images were obtained using a Dragonfly 503 spinning disk confocal microscope using software Fusion (v.2.1.0.80) (Andor, Oxford Instruments, Abingdon, UK) with a 20× objective and the camera Zyla 4.2 PLUS sCMOS using a 40 μm pinhole size. The 3D and 2D projections were created using IMARIS Viewer software (v.10).

### Liquid Chromatography-Mass Spectrometry (LC-MS) Sample Preparation

The cultivation media of all 3D and 2D control and minoxidil-treated samples (the number of analyzed samples is specified in the figure legends) were collected and dialyzed for 5 days at 20 °C. The dialysis solution (0.01% NaN₃ in distilled water) was replaced 5 times. Samples were solubilized in 1% (w/v) SDS in 100 mM TEAB (triethylammonium bicarbonate), sonicated and processed according to the solvent precipitation (SP4) no glass bead protocol [33]. Briefly, samples were reduced with 10 mM TCEP (tris(2-carboxyethyl)phosphine), alkylated with 40 mM CAA (chloroacetamide), performed together at 95 °C for 10 min, and digested with trypsin (Trypsin Gold, Mass Spectrometry Grade, Promega V5280) overnight at 37 °C at a 1:50 ratio (trypsin:protein).

### LC-MS Analysis and Data Processing Protocol

Samples were desalted on Empore C18 columns, dried in Speedvac, and dissolved in 0.1% trifluoroacetic acid + 2% acetonitrile. 500 ng of desalted peptide digests were separated on a C18 column using a 60 min elution gradient (Dionex Ultimate 3000, flow rate 300 nL/min) and analyzed in data-independent acquisition (DIA) mode on an Orbitrap Exploris 480 mass spectrometer equipped with a FAIMS unit (Thermo Fisher Scientific) set to CV-45V. DIA MS raw files were processed in Spectronaut (v.19.9, Biognosys) using direct DIA mode and human proteome UP000005640_9606.fasta (UniProt release 2025_01) and a default setting with Precursor and Protein Q-value and PEP cutoff set at 0.01. Downstream data processing was performed using Perseus software (v.2.1.4.0). Protein data were log2-transformed. A t-test was used to analyze protein expression, with a permutation-based false discovery rate (FDR) correction (S0 = 0.1 and FDR = 0.05) and 250 randomizations. Data are averaged from at least three independent biological replicates (n ≥ 3) in each group. For principal component analysis (PCA), missing values were imputed using a normal distribution. Protein interaction network analysis was performed using Search Tool for the Retrieval of Interacting Genes (STRING) database (v.12.0) [34] with a 0.4 confidence threshold. K-means clustering was performed to cluster significantly changed proteins. The mass spectrometry proteomics data have been deposited to the ProteomeXchange Consortium via the PRIDE [35] partner repository with the dataset identifier PXD072354.

### Collagen Type I Production Analysis

Soluble collagen was measured in the media conditioned by the cells cultured with/without 0.5 mM minoxidil for 3 days after the last media change until the end of the experiment. Samples (n = 12) were dialyzed and lyophilized as described above. Half of each sample was dissolved in 6 N HCl and digested at 105 °C for 3 h and subjected to total soluble collagen quantification using the Hydroxyproline Colorimetric Assay Kit (Sigma-Aldrich, MAK008), performed according to the manufacturer’s instructions. The second halves of the samples were subjected to 8% polyacrylamide gel electrophoresis. The stained gels were scanned with an imaging densitometer GS-800 (Bio-Rad), and protein bands were quantified by Quantity One software (Bio-Rad, v.4.6.8) (Additional file 6). Identification of the collagen type I band was confirmed by mass spectrometry.

### Statistical Analysis

With the exception of the analysis of LC-MS data, GraphPad Prism 10 was used for the statistical test. All data were tested for normality using Shapiro-Wilk´s test. Paired t-tests were used (or a Wilcoxon paired test if normality and equal variance of the data were not met). All plots (except for LC-MS data) were created in GraphPad Prism. Graphs show individual values, the line at median, the 25th to 75th percentiles and the minimum and maximum values, if not stated otherwise. Statistically significant p-values are indicated in the figures: *p < 0.05, **p < 0.01.

## Results

### Decellularization

**Fig. 1:**
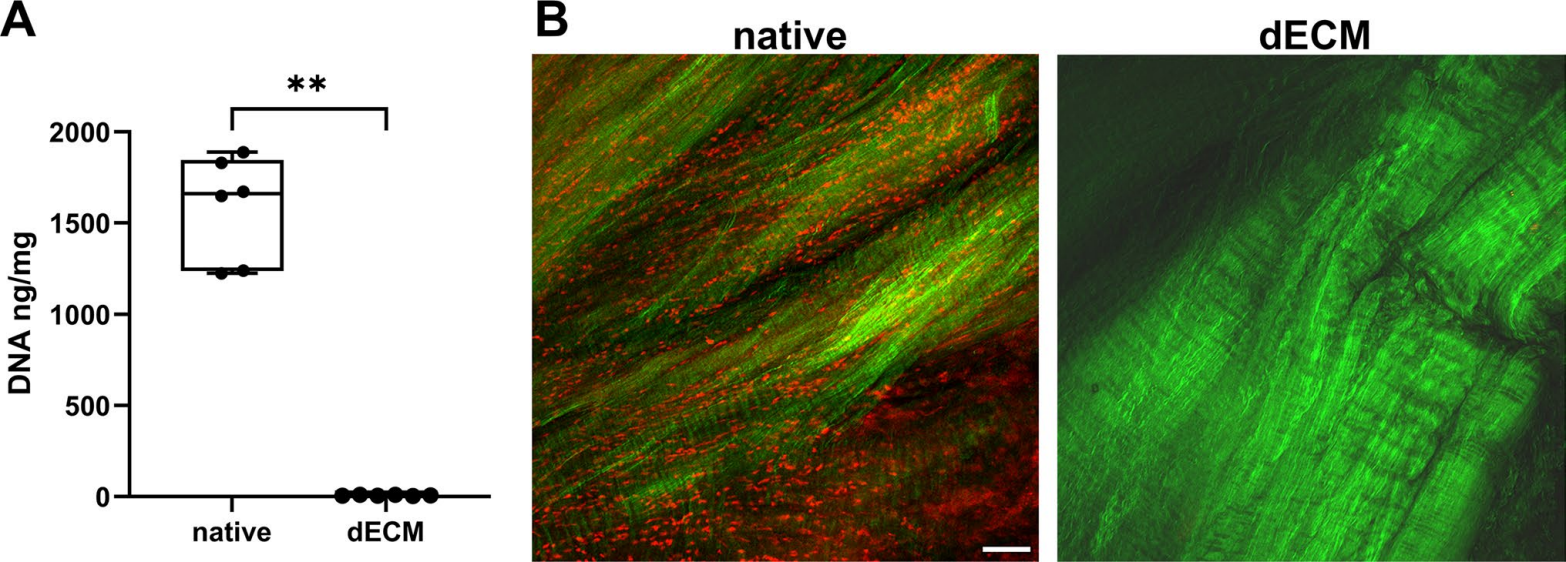
**A** DNA content in native and dECM samples measured by PicoGreen assay. Paired t-test, ** p ≤ 0.01. **B** Nuclear Draq5 DNA staining (red) and SHG signal of collagen fibers (green). Scale bar = 50 µm. Maximum intensity projections

The efficiency of cell removal was quantified by measuring the residual DNA. All the decellularized samples tested returned levels below 50 ng DNA per mg of dry tissue, which is generally accepted as the maximum level of DNA present in decellularized tissue [36] (Fig. 1A). The decellularized samples stained with Draq5 had no positive signal from nuclei compared to the native samples. The SHG microscopy provided comparative imaging of native and decellularized sample groups, revealing normal structural appearance and standard collagen fiber quality (Fig. 1B). A protocol using 0.5% SDS in TE buffer of pH 8 with protease inhibitors provided optimal decellularization, balancing effectiveness and tissue preservation. In contrast, when 1% SDS and 1% Triton X-100 solutions were tested, the former caused significant protein loss and collagen denaturation. At the same time, the latter failed to remove the DNA and cellular debris adequately (Additional file 2).

### Characterization of dECM Scaffold

**Fig. 2:**
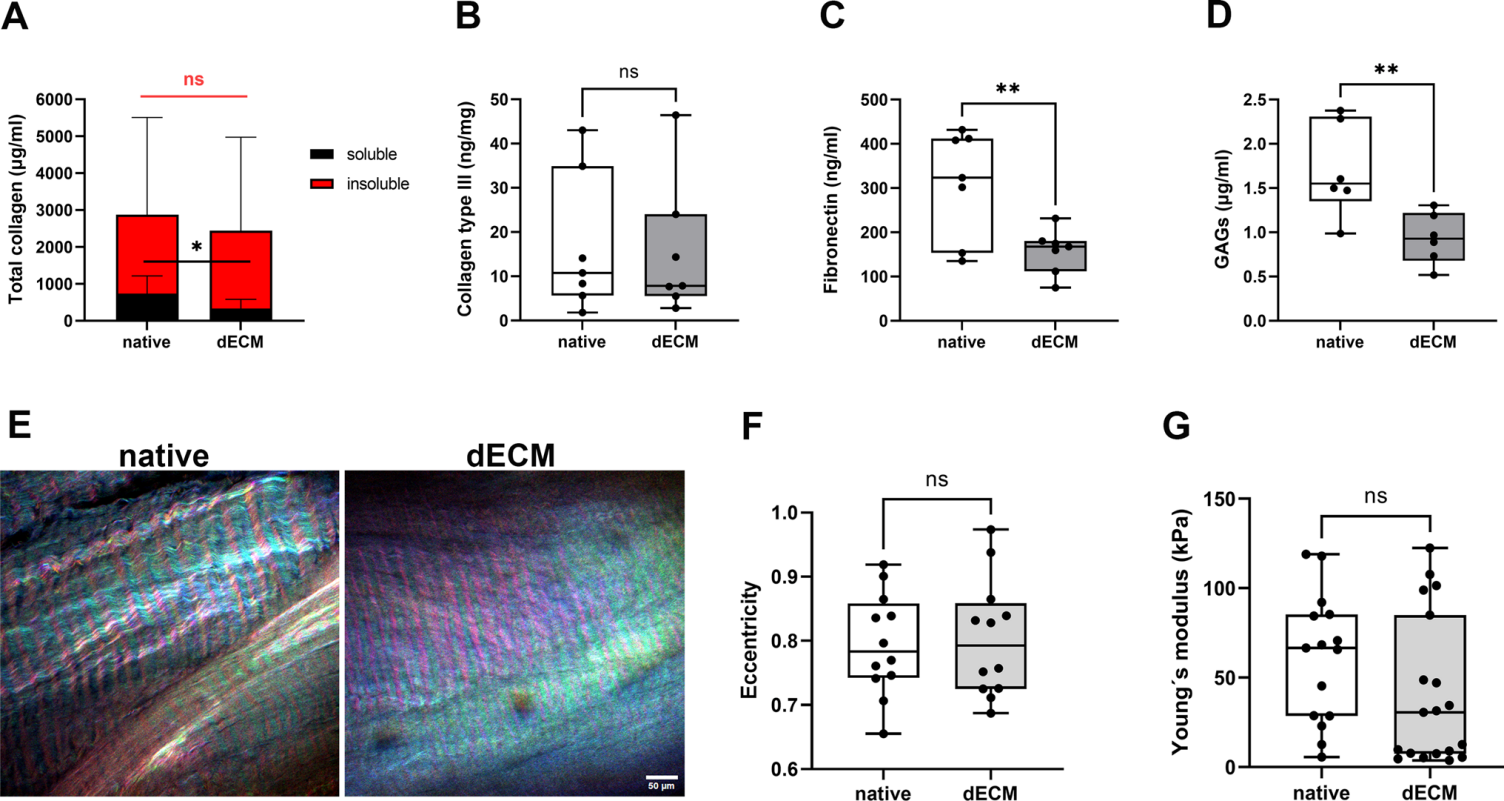
Characterization of the dECM scaffold: **A** Soluble/insoluble collagen levels, stacked column plot, mean + SD, paired t-test for soluble collagen, Wilcoxon nonparametric paired test for insoluble collagen. **B** Collagen type III, Wilcoxon nonparametric paired test. **C** Fibronectin, paired t-test. **D** Glycosaminoglycans, paired t-test. **E** Overlay of SHG signal. Colors correspond to the dominant polarization angle: 0° (Red), 60° (Green), and 120° (Blue). Variations in color saturation indicate the relative isotropy of the signal. Scale bar = 50 µm. **F** Analysis of fiber orientation. Paired t-test. **G** The indentation test results are represented by Young’s modulus, Wilcoxon paired nonparametric test. Statistical significance *p ≤ 0.05; ** p ≤ 0.01; no statistical significance (ns)

To assess the main components of the resulting dECM scaffold, we quantified the total soluble and insoluble collagen content, type III collagen, fibronectin, and glycosaminoglycans (GAGs) to ensure that the scaffold retains its structural and bioactive functionality (Fig. 2A–D). The level of insoluble (i.e., crosslinked) collagen did not significantly decrease in the decellularized samples compared to the native samples (p = 0.843). Similarly, the level of type III collagen, which is strongly associated with fibrotic processes [37, 38], also remained unchanged (p = 0.813). However, we found a significant decrease in the level of soluble collagen in dECM compared to the native matrices (p = 0.011), fibronectin (p = 0.002), and glycosaminglycans (p = 0.001). The SHG and pSHG microscopy images provided information on collagen fiber quality, local collagen fiber orientation, and global collagen fiber alignment (Fig. 2E, F). The presence of the SHG signal in the decellularized samples indicated that the fibrillar structure of the collagen appears to be minimally affected during the process of decellularization. The pSHG analysis of the fiber orientation was expressed in terms of eccentricity, where an eccentricity of $\upvarepsilon =0$ indicates a strongly isotropic orientation, which is typically observed for scattered or broken fibers. In contrast, $\upvarepsilon =1$ indicates a very strong anisotropic line-like structure in the tissue, with fibers aligned in one direction. The analysis showed no significant difference (p = 0.857) between the native and decellularized samples, demonstrating one predominant direction of collagen fibers and the unchanged fiber orientation after decellularization. The mechanical properties of dECM scaffolds were compared to native tissue sections. There was no statistically significant difference (p = 0.083) between Young´s moduli of native and dECM samples with noticeable inter-individual variability (Fig. 2G).

### dECM Recellularization

**Fig. 3:**
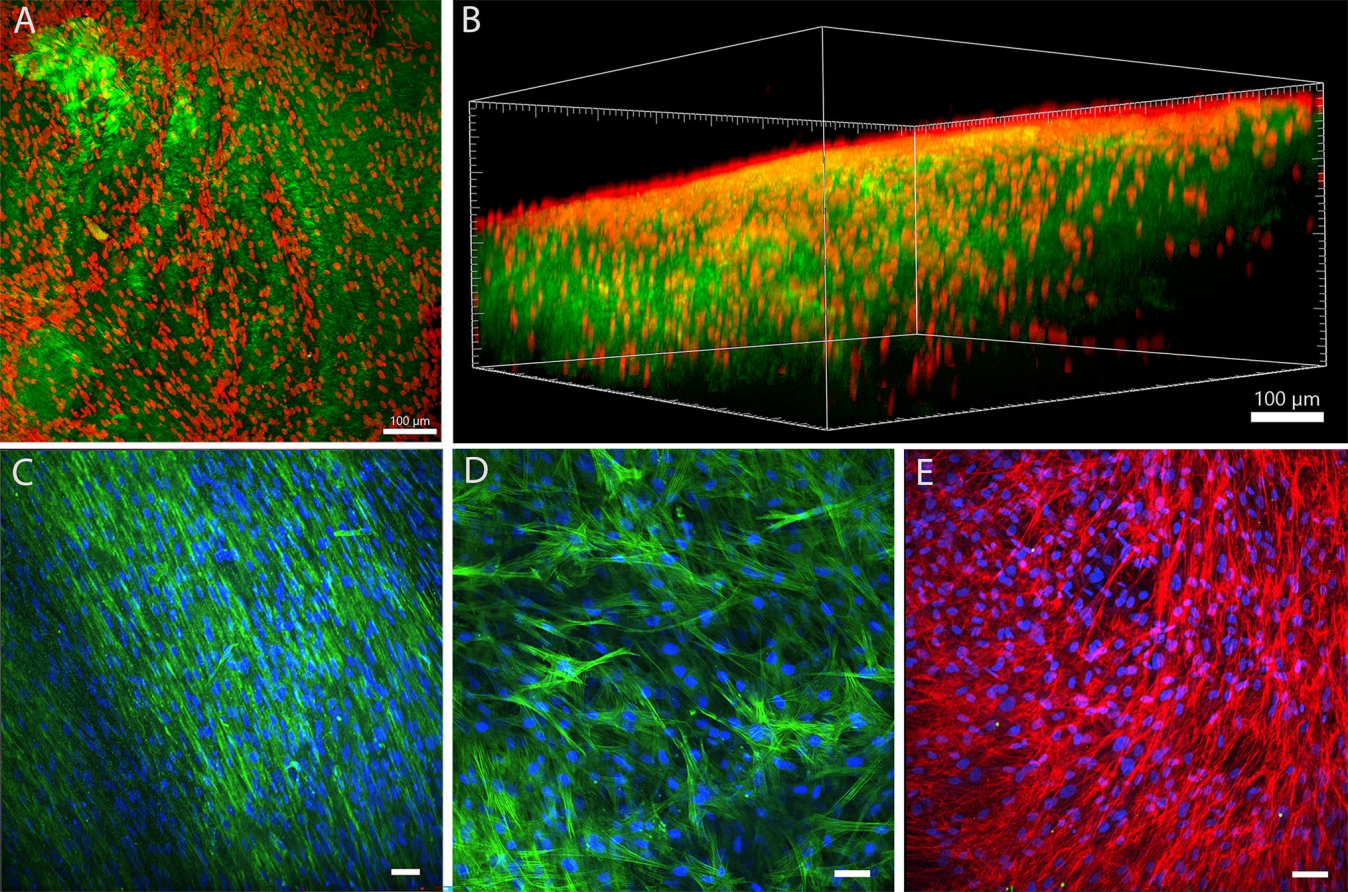
Visualization of the 3D in vitro model of DD fibrosis, i.e., human dECM loaded with fibroblasts after 3 weeks of culture; top view (**A**) and side view (**B**), Cell nuclei are stained in red; SHG signal of collagenous dECM in green, scale bar = 100 µm, maximum intensity projections. **C** Immunofluorescence staining of collagen type I (green), nuclei counterstained in blue, maximum intensity projection. **D** Immunofluorescence staining of αSMA fibers (green), nuclei are counterstained in blue, a horizontal section. **E** Immunofluorescence staining of cellular ED-A fibronectin domain (red), cell nuclei counterstained in blue, maximum intensity projection; C-E: scale bar = 50 µm

Seeding the dECM with human fibroblasts, derived from DD tissue, and their subsequent infiltration into the matrix constitutes the 3D in vitro model of DD (Fig. 3A, B). Initial attempts to culture DD fibroblasts on dECM in standard DMEM with 10% of FBS resulted in poor cell proliferation, low matrix invasion, and minimal new collagen production (Additional file 3). To improve these outcomes, a stepwise cultivation approach was introduced. First, in the proliferative phase, the cells were cultured for one week on dECM in DMEM supplemented with 5% of human platelet lysate and with ascorbic acid to promote proliferation. Platelet lysate is rich in a wide variety of growth factors and is known to enhance cell expansion [39, 40]. Second, the medium was changed to DMEM with a low concentration of FBS (2%) and 2.5 ng/ml of TGFβ1 and ascorbic acid to induce the differentiation of fibroblasts into contractile myofibroblasts. The production of newly synthesized collagen type I was visualized by immunostaining (Fig. 3C). The myofibroblastic phenotype was confirmed by immunostaining of α-SMA assembled into the fibers (Fig. 3D), and also by the presence of cellular domain A of fibronectin (ED-A fibronectin; Fig. 3E), which is crucial for the induction of myofibroblastic phenotype by TGFβ1 [41, 42]. It should be emphasized that repeated degassing of the dECM, first before laminin coating and then again before seeding which removes trapped air bubbles, thereby creating space for cells, together with long cultivation (3 weeks), led to a more effective penetration of cells into the matrix. Interestingly, even before TGF-β1 stimulation, ED-A fibronectin fibers were already present. Thin α-SMA fibers were also detectable in some cells, though not uniformly across the samples (data not shown). This suggests the contribution of ECM to the myofibroblast phenotype but it also points to substantial matrix heterogeneity of the samples.

### Disease Relevance of the 3D Model

**Fig. 4:**
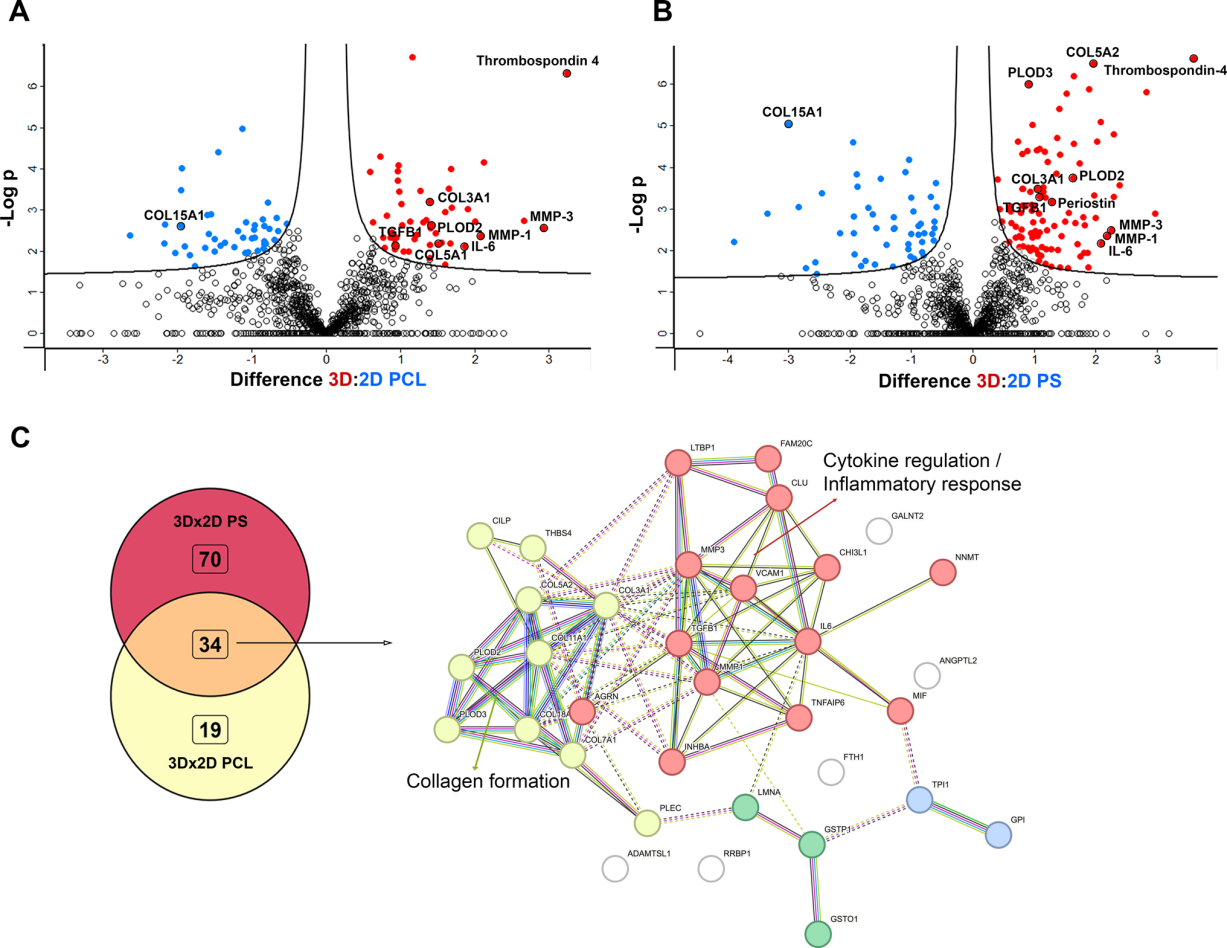

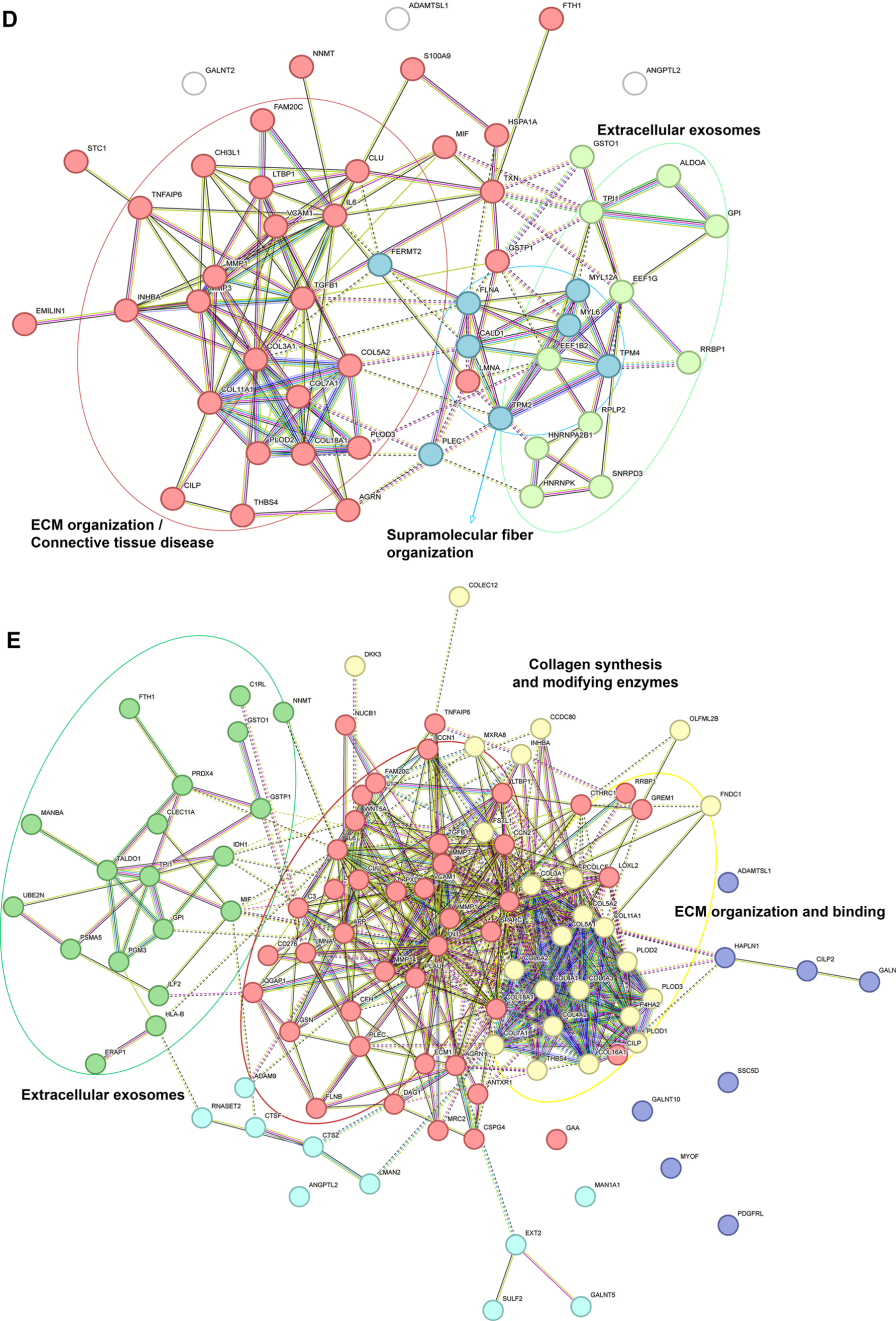
Comparison of secretomes of cells cultivated on either 3D dECMs (3D) (n = 6), polycaprolactone nanomembrane (2D PCL) (n = 3), or polystyrene well-plate (2D PS) (n = 3). Volcano plots show significantly upregulated (red, right side) and downregulated (blue, left side) proteins in 3D samples compared to 2D PCL (**A**) and 2D PS (**B**). The X-axis represents protein difference (log2-transformed fold change), and the Y-axis the corresponding -log10-transformed p values. **C** Venn diagram shows the number of unique and shared proteins upregulated in 3Dx2D PCL and 3Dx2D PS comparisons. The overlap of 34 proteins upregulated in 3D samples is visualized by STRING protein-protein interaction analysis. All proteins that were upregulated in 3D samples compared to 2D PCL samples (**D**) and 2D PS samples (**E**). Intracluster interactions are shown in solid lines, intercluster interactions are shown with dotted lines

To evaluate the relevance of our 3D in vitro construct for modeling of DD, we compared the secretomes of cells cultured on the 3D dECM scaffolds with the secretomes of cells cultured on conventional 2D surfaces polycaprolactone nanofiber membranes (referred to as 2D PCL) and polystyrene well-plates (referred to as 2D PS). The principal component analysis (PCA) of the data revealed distinct clusters that can distinguish the 3D samples from 2D PCL and 2D PS samples, respectively (Additional file 4, Fig. S1). The volcano plots (LC-MS results) show significantly changed protein concentration between 3D and 2D PCL (Fig. 4A), and between 3D and 2D PS samples, respectively (Fig. 4B), highlighted are proteins typically involved in fibrotic disease and tissue remodeling e.g.: type III collagen, TGFβ-1 proprotein, matrix metalloproteinases 1 and 3 (MMP-1 and MMP-3), lysyl hydroxylase 2 (coded by PLOD2 gene), interleukin 6, thrombospondin 4 and periostin. Importantly, we detected the unprocessed full-length TGF-β1 proprotein, suggesting that it is endogenously produced by the fibroblasts. (The TGF-β1 peptide added to the differentiation medium, however, was the active, mature form). Interestingly, cells cultivated on 2D PS produced significantly more type I collagen compared to 3D cultivation, which we interpret as a result of a non-physiological, ultra-stiff (GPa) and ECM-deficient environment rather than of fibrotic stimuli. The Venn diagram shows the overlap of proteins upregulated in the 3D cell secretome compared to 2D PCL and 2D PS secretomes. Thirty-four proteins were found to be upregulated in the 3D secretome data set relative to both 2D samples (Fig. 4C). The STRING protein-protein interaction analysis of these 34 proteins shows 2 main clusters: one associated with cytokine regulation and inflammatory response (including interleukin 6 (IL-6), TGFβ1 proprotein, latent transforming growth factor-β binding protein-1 (LTBP1), macrophage migration inhibitory factor (MIF) and others) and the second cluster related to collagen formation (containing different types of collagens and collagen processing enzymes). The two clusters are interconnected primarily via MMPs and TGFb1 proteins. Fig. 4C and D show the STRING analysis of all significantly upregulated proteins in 3D samples compared to 2D PCL (Fig. 4C) and 2D PS, respectively (Fig. 4D). Both comparisons revealed clusters of proteins associated with collagen synthesis, ECM organization and binding, increased exosome secretion, and supramolecular fiber organization. Additional file 4, Tables S1 and S2 provides the complete list of the significantly upregulated and downregulated proteins in 3D samples compared to 2D PCL and 2D PS, resp.

### The Antifibrotic Effect of Minoxidil

**Fig. 5:**
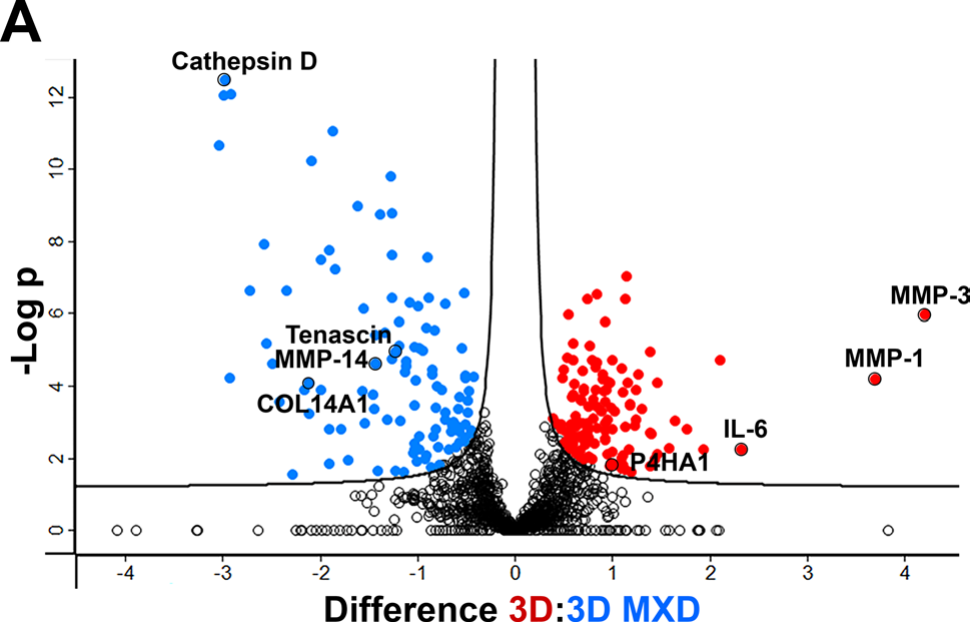

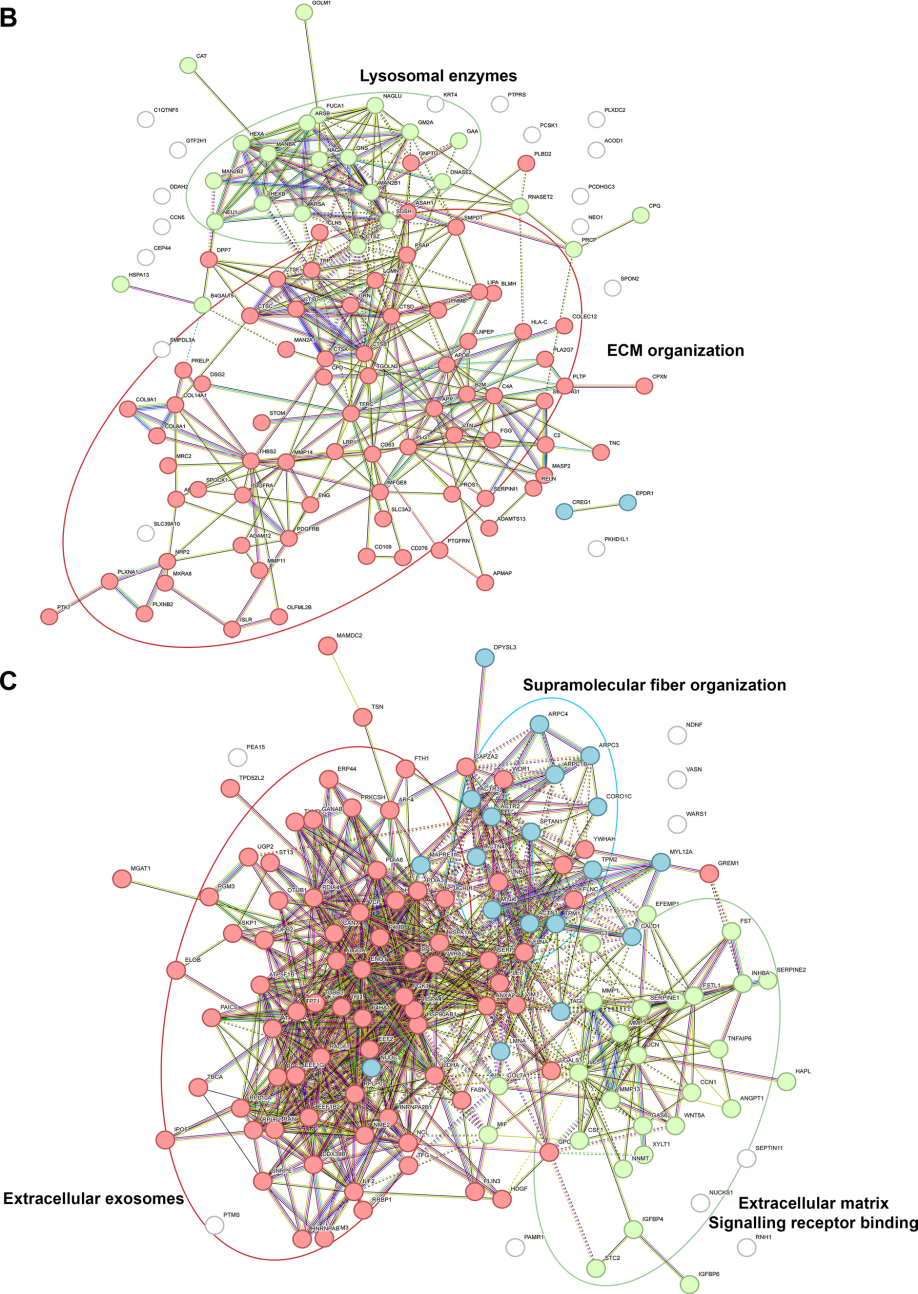

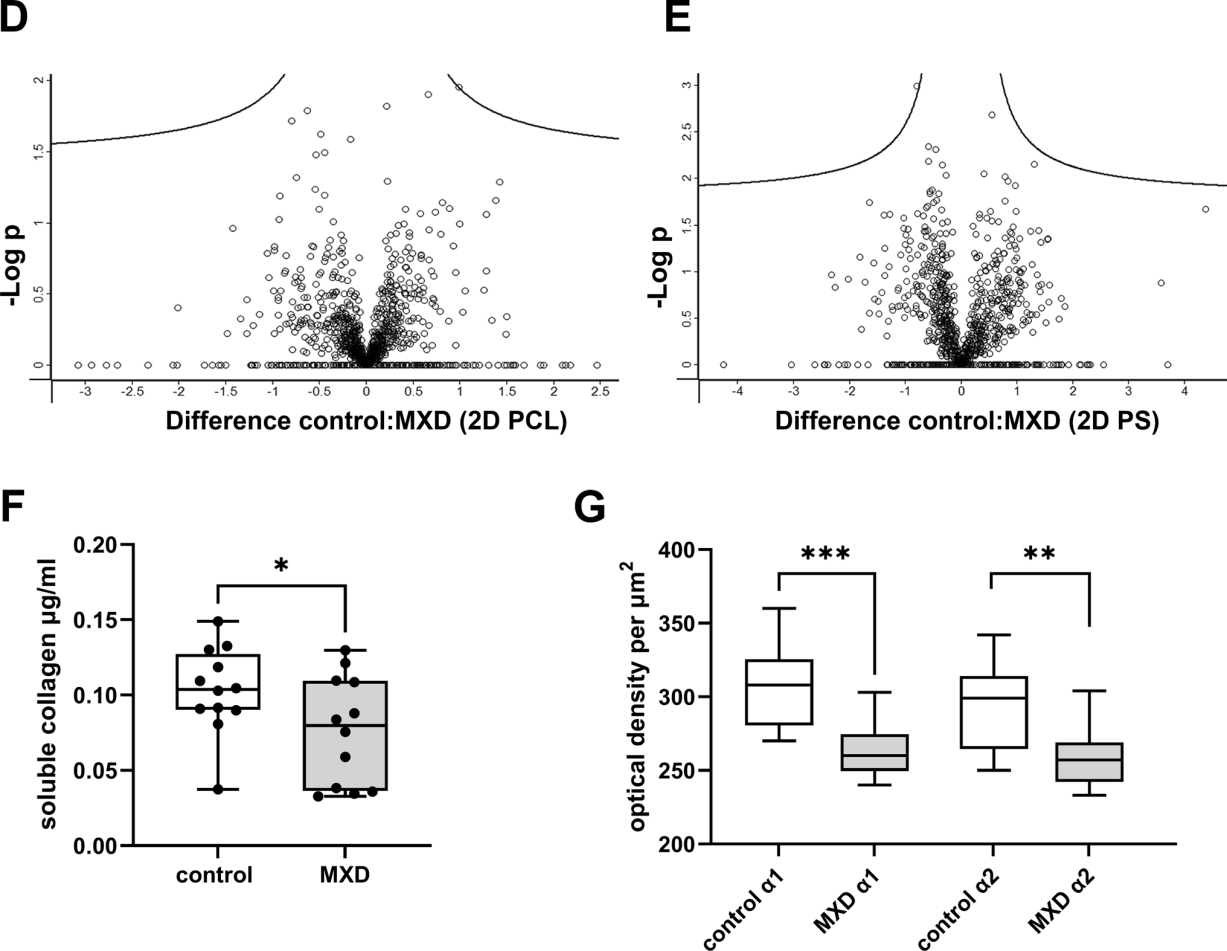
The effect of minoxidil. **A** The Volcano plot shows upregulated (red, right side) and downregulated (blue, left side) proteins in 3D control samples compared to 3D MXD-treated samples (n = 9). **B** STRING protein-protein interaction analysis of proteins upregulated (**B**) or downregulated (**C**) in 3D MXD-treated samples. Intracluster interactions are shown in solid lines, intercluster interactions are shown with dotted lines (**D**). Volcano plot shows no significantly changed proteins in 2D PCL or **E** 2D PS control and MXD-treated samples (n = 3). The X-axis represents protein difference (log2- transformed fold change), and the Y-axis the corresponding -log10-transformed p values. **F** Total soluble collagen levels in MXD-treated samples compared to control samples measured by the hydroxyproline assay, paired t-test. **G** Densitometry analysis of electrophoretic bands of individual α1, α2 collagen type I chains, paired t-tests. Statistical significance: *p ≤ 0.05; **p ≤ 0.01; ***p ≤ 0.005; no statistical significance (ns)

We used 0.5 mM minoxidil (MXD) to test and optimize analytical methods for accurately quantifying differentially expressed proteins relevant to drug screening and fibrosis research. This concentration had no significant cytotoxic effect on cell viability as measured by the standard resazurin metabolic assay (data not shown). MXD is an inhibitor of lysyl hydroxylases, enzymes involved in collagen crosslinking and often upregulated in fibrotic tissues, including DD [43, 44]. We performed proteomic analysis of the secretome of control and MXD-treated samples cultured on 3D dECMs as well as on both 2D surfaces. The resulting volcano plot shows 251 significantly changed proteins when cells were cultivated on 3D dECM (Fig. 5A) and no significantly changed protein when cells were cultivated on either 2D nanofiber membrane or polystyrene well-plate (Fig. 5D, E). For 2D surfaces, only 3 samples were analyzed, as these are homogeneous and highly reproducible. In contrast, the 3D samples exhibited heterogeneity among patient-derived dECM scaffolds; therefore, 9 samples were used. Nevertheless, even when analyzing only 3 × 3 samples in a 3D control x 3D MXD experiment, a total of 131 significantly altered proteins were observed (Additional file 5, Fig. S1). In Fig. 5A we highlight the proteins whose quantification may be relevant to fibrotic processes. The complete list of significantly regulated proteins in 3D control versus MXD-treated samples is provided in Additional file 5, Table S1. The principal component analysis of the data revealed distinct clusters that distinguished 3D and 3D MXD-treated samples (Additional file 5, Fig. S2). The STRING protein-protein interaction analysis of significantly changed proteins in 3D control and MXD-treated cells showed that minoxidil induces changes in ECM organization and lysosomal stress as suggested by the upregulation of lysosomal enzymes (Fig. 5B). STRING analysis of proteins downregulated in MXD-treated samples showed decreased extracellular exosome secretion and complex changes in ECM**.** Although proteomic analysis can quantify most proteins, it is less suitable for the proteins poorly digested by trypsin, such as fibrillar type I collagen. For its more accurate measurement, we optimized SDS-based electrophoresis and the hydroxyproline assay to detect this protein released to the cultivation medium within 3 days at the end of the experiment. The total soluble collagen levels were significantly decreased in MXD-treated samples (p = 0.017) as quantified by hydroxyproline assay (Fig. 5F). We also analyzed individual collagen type I chains (i.e., α1, α2) by SDS gel electrophoresis. A significant decrease of α1 and α2 chains was observed in the MXD-treated samples (p = 0.0001 and p = 0.001, respectively) (Fig. 5G). The addition of 0.5 mM MXD did not reverse the differentiation of myofibroblasts; the immunofluorescence images of ED-A FN or α-SMA fibers were similar to the control samples (data not shown). These results imply that MXD induced changes in collagen processing, leading to reduced collagen production and a subsequent complex reorganization of the extracellular matrix (ECM), accompanied by cellular stress and an overall decrease in cell secretion (Fig. 5).

## Discussion

This study aimed to develop a physiologically relevant and reproducible 3D in vitro model that preserves the DD-specific microenvironment by combining decellularized patient-derived ECM-based scaffolds with patient-derived fibroblasts. To the best of our knowledge, no such model has yet been created. We provide the detailed protocol for assembling the dECM scaffold with the DD cells supporting cell infiltration into the dECM and reactivation of fibrotic behavior in the otherwise quiescent fibroblasts. Fibrotic tissue in Dupuytren’s disease is highly heterogeneous between patients. This heterogeneity is preserved in decellularized ECM, which retains differences in collagen composition (collagen types I and III), GAG content, and mechanical properties such as stiffness and fibrillar architecture. Consequently, some dECM samples may provide a stronger profibrotic microenvironment than others. To establish a robust and reproducible 3D model of Dupuytren’s disease that reliably recapitulates key fibrotic hallmarks, we therefore employed a step-wise stimulation strategy. This approach involves an initial phase of enhanced proliferation using platelet lysate, followed by controlled induction of myofibroblast differentiation via TGF-β stimulation.

One of the most advanced models of DD published to date is an ex vivo system that enables the study of the complex fibrotic microenvironment of DD. It allows clinically relevant drug testing and cell-cell and cell-matrix studies but is limited to short-term. The tissue typically remains viable for only 2 days, and with an advanced incubation system, for 7 days and requires access to fresh surgical tissue and its immediate processing [22, 23]. In comparison, our DD in vitro model allows for tissue and cell storage prior to the onset of experiment as well as longer cultivation periods (typically 21 days) and repeated continuous testing of the substance’s effect using secretome analysis. In a future study, it will be important to focus on the analysis of the newly deposited matrix, although it is methodologically challenging to distinguish the matrix forming the dECM from the newly deposited matrix.

Numerous studies highlight the importance of both tissue-specific cells and ECM for 3D in vitro DD modeling as cell behavior is inseparable from the ECM context. The ECM directly drives the disease progression through biochemical and biomechanical feedback (reviewed in [45]). The most recent proteomic study of DD ECM [20] shows that the main functions of upregulated proteins are associated with matrix metalloproteinase activation, trimerization of collagen chains, and ECM organization, suggesting excessive ECM remodeling. Similarly, cells in our 3D model release proteins that were clustered into groups: collagen synthesis; ECM organization and, interestingly, extracellular exosomes (Fig. 4). Fibrosis has been increasingly linked to changes in extracellular vesicle dynamics. Recent studies indicate that fibrotic tissue actively promotes or is associated with increased extracellular vesicle secretion [46–48].

Not only biochemical composition but also matrix biomechanics directly influence cellular responses. Fibrotic tissue are typically found to be stiff, with Young’s moduli of 20–100 kPa, (reviewed in [49, 50]). Layton et al. reported the Young’s modulus of DD nodules at around 9 kPa [51] with large variability across individual nodules. Our results of native DD tissue showed Young´s modulus ranging from 5 to 119 kPa and confirmed the heterogeneity of nodular tissue (Fig. 2G). Viji Babu et al. [52] reported that Dupuytren’s fibroblasts exhibit increased stiffness and α-SMA expression compared to normal and scar fibroblasts, especially in response to TGF-β1, highlighting their active fibrotic phenotype. Importantly, their other study [53] also showed that fibroblasts dynamically interact with the ECM, influencing and being influenced by matrix stiffness and architecture. This is consistent with our proteomic data; we demonstrate that DD fibroblasts cultivated in a 3D in vitro dECM-based model exhibited a disease-relevant secretory phenotype compared to DD fibroblasts cultivated on a stiff 2D PCL membrane (Young’s moduli ~ MPa, 10-times higher than dECM) [54, 55] (Fig. 4A) or the still commonly used ultra-stiff tissue culture polystyrene (~ GPa, 10, 000-times higher than dECM and any fibrotic tissue in vivo) [56] (Fig. 4B). This indicates that not just stiffness but a combination of different local physical and biomechanical stimuli, such as roughness, topography, or fiber alignment, are sensed by cells (reviewed in [57]). Previous transcriptomic analyses of DD fibroblasts have shown that 2D or 3D cultivation strongly influences the detection of disease-related gene expression. While Satish et al. [58] identified 40 of differentially expressed genes between DD and carpal tunnel fibroblasts under standard monolayer culture conditions, a subsequent study by the same group demonstrated that culturing fibroblasts on a collagen layer revealed a substantially more (894) differentially expressed genes [59]. These studies highlight the importance of collagenous matrix environment in modulating fibroblast gene expression. Similarly, our results on the effects of MXD on cells cultured on 2D and 3D substrates show that interactions with 2D substrates are less complex than those with 3D substrates (Fig. 5).

Early investigations identified myofibroblasts as a dominant cell type in DD nodules, accompanied by M1 pro-inflammatory macrophages [14]. Recent works by Gonga- Cavé et al. [21] and Heinmäe et al. [20] further clarify that DD fibroblasts or DD-derived ECM can independently activate macrophages, which in turn drive fibroblast migration and myofibroblast differentiation through paracrine cytokine signaling. In our study, instead of cell paracrine stimulation, the active form of TGF-β1 was added into the medium. Although TGF-β1 was added to all experimental groups, 3D and 2D, a significant increase in proteins associated with TGF-β (e.g. TGF-β proprotein) and inflammatory signaling (e.g. IL-6) was observed only in the 3D samples (Fig. 4).

The incomplete understanding of the pathogenesis of DD represents a significant challenge to the development of effective antifibrotic therapies. DD is not a life-threatening condition, and as such, it is unlikely that this form of fibrosis will be prioritized in the development of therapies. For pulmonary or liver fibrosis, extensive research has identified targeted treatments (reviewed in [60]). The knowledge from other fibrotic and inflammatory diseases allows drug repurposing if an appropriate DD-relevant model is available. We tested the antifibrotic effect of minoxidil in the 3D in vitro model described using an efficient and accessible secretome analyses (Fig. 5). The active substance minoxidil, widely known for its beneficial effects on hair loss, is an inhibitor of collagen crosslinking. Its inhibitory effect on collagen deposition and pseudo-3D hydrogel shrinkage has been described by our team in experiments on clubfoot-derived cells [27, 61]. The current study shows a significant decrease of soluble collagen type I, prolyl-4 hydroxylase and IL-6, MMP-1, and MMP-3 in 0.5 mM minoxidil-treated cells while elevating their lysosomal enzymes (Fig. 5), suggesting lysosomal stress, which is not detectable by conventional viability assays.

Although the new 3D in vitro model of DD resembles DD tissue, we recognize that the proposed model has some limitations, namely substantial heterogeneity of dECMs reflecting the biological variability and a disease-state between patients. While the decellularized scaffold provides structural cues, it lacks the dynamic mechanical stimulation and immune components that are present in vivo. Future improvements could include incorporating mechanical loading and/or co-culture of fibroblasts with immune cells to better reflect the disease microenvironment.

## Conclusion

We successfully established a new 3D in vitro model of DD based on dECM repopulated with DD-derived fibroblasts. The decellularized dECM had defined structural and functional properties. These include collagen fiber quality, orientation, and mechanical stiffness of the dECM, while maintaining key characteristics of native tissue, although a varying degree of reduction of the matrix components was observed.

The dECM was effectively repopulated with DD-derived fibroblasts, while optimized culture conditions supported cell proliferation, migration, and differentiation into myofibroblasts. The soluble collagen and proteomic analysis of secretome proved that the secretory profile of DD cells cultivated on 3D dECM was more disease-specific (profibrotic) than on both the 2D PCL membrane and 2D polystyrene surfaces. To date, the in vitro secretome of DD fibroblasts has not been so specifically investigated by proteomics.

The evaluation of the antifibrotic effect of minoxidil validated the functionality and effectiveness of our 3D in vitro model as a platform for drug screening. Both 2D PCL and 2D PS materials seeded with DD fibroblasts failed to show a significant response to minoxidil treatment.

Overall, our 3D in vitro model of DD provides a disease-relevant and reproducible culture system suitable not only for preclinical analysis but also for the investigation of cell and ECM contributions to fibrotic processes.

## Supplementary Information

Below is the link to the electronic supplementary material.

Characterization of polycaprolactone nanofibers

Comparison of three decellularization treatments

Comparison of standard and stepwise cultivations

Proteomic data. PCA analysis and the list of significantly regulated proteins secreted by the cells cultivated on 3D dECM scaffolds

Proteomic data. PCA analysis of secretomes of control and minoxidil treated 3D samples. The list of significantly regulated proteins secreted by the cells cultivated on 3D dECM scaffolds with/without minoxidil

Analysis of polyacrylamide SDS gel electrophoresis

## Data Availability

The datasets used and analyzed during the current study are available from the corresponding author on reasonable request.
