# Supplementary material for: Development of a 3D In Vitro Model of Dupuytren’s Disease as a Platform for Drug Screening
Source: Cell Mol Bioeng. 2026 Jan 19;19(1):111–27. doi: 10.1007/s12195-026-00885-2 (PMC13031596; doi:10.1007/s12195-026-00885-2)
Supplement: Supplementary file 1 — Characterization of polycaprolactone nanofibers [file 12195_2026_885_MOESM1_ESM.pdf]

Additional file 1

Title: Characterization of polycaprolactone (PCL) nanofibers

The morphology of the prepared PCL membrane was analyzed from images obtained by a scanning electron microscope (SEM) (Vega S3B EasyProbe, Tescan Orsay Holding a.s., Czech Republic). The average fiber diameter was counted from 300 measurements using ImageJ software (v.1.54k). The PCL membrane was produced with an average fiber diameter 1.33 ± 0.93 μm. The average areal weight of material was 40 g/m^2.

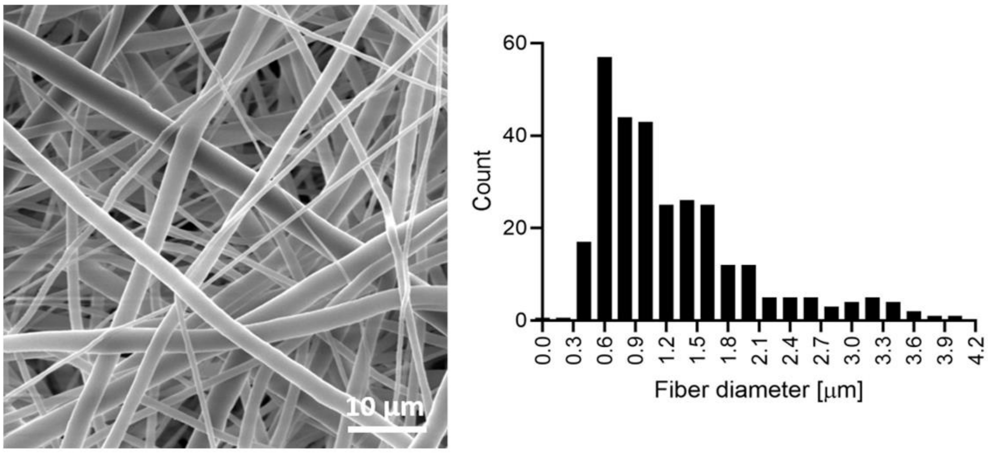

Fig. S1.: Representative SEM picture and the corresponding histogram of fiber diameter distributions; scale bar = 10 μm.

Article title:

“Development of a 3D *in vitro* model of Dupuytren’s Disease as a platform for drug screening”

Journal name:

Cellular and Molecular Bioengineering

Author names:

Jarmila Knitlova, Adam Eckhardt, Daniel Hadraba, David Vondrasek, Roman Stachon, Elena Filova, Vera Jencova, Kristyna Havlickova, Tatyana Kobets, Martin Ostadal and Lucie Bacakova

Affiliation:

Laboratory of Translational Metabolism,
Institute of Physiology of the Czech Academy of Sciences,
Videnska 1083, 142 00 Prague 4, Czech Republic;
+420 724 066 868

e-mail address of the corresponding author:

adam.eckhardt@fgu.cas.cz
