# Supplementary material for: Development of a 3D In Vitro Model of Dupuytren’s Disease as a Platform for Drug Screening
Source: Cell Mol Bioeng. 2026 Jan 19;19(1):111–27. doi: 10.1007/s12195-026-00885-2 (PMC13031596; doi:10.1007/s12195-026-00885-2)
Supplement: Supplementary file 2 — Comparison of three decellularization treatments [file 12195_2026_885_MOESM2_ESM.pdf]

Additional file 2

Title: Comparison of three decellularization treatments

The second harmonic generation (SHG) microscopy with Draq5 DNA staining provided comparative imaging of the patient-derived matrix exposed to 0.5% SDS, 1% SDS, or 1% Triton for 2 hours. We observed complete DNA removal with standard collagen fiber quality using a 0.5% SDS solution. In contrast, 1% SDS solution resulted in a blurred and reduced SHG signal, indicating structural disruption. Treatment with 1% Triton X-100 showed incomplete DNA removal, although collagen fiber quality remained standard. Details on DNA treatment and washing procedures are provided in the "Decellularization" section of the Materials and Methods. Acquisition and staining correspond to **Fig. 1B** in the manuscript.

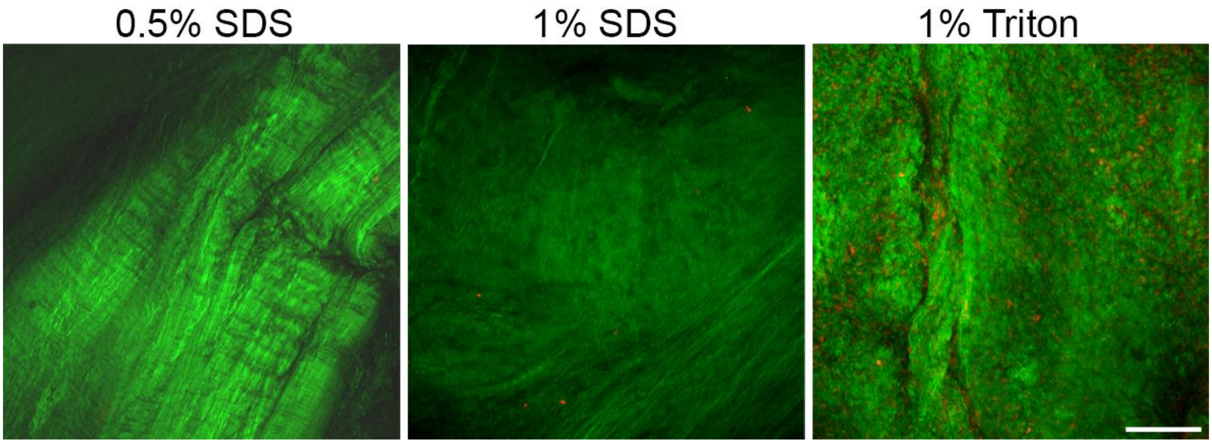

Fig. S1: Comparison of three decellularization treatments. Green: SHG signal of collagen type 1, red: nuclear Draq5 staining. Scale bar = 100 µm.

Article title:

"Development of a 3D *in vitro* model of Dupuytren's Disease as a platform for drug screening"

Journal name:

Cellular and Molecular Bioengineering

Author names:

Jarmila Knitlova, Adam Eckhardt, Daniel Hadraba, David Vondrasek, Roman Stachon, Elena Filova, Vera Jencova, Kristyna Havlickova, Tatyana Kobets, Martin Ostadal and Lucie Bacakova

Affiliation:

Laboratory of Translational Metabolism,
Institute of Physiology of the Czech Academy of Sciences,
Videnska 1083, 142 00 Prague 4, Czech Republic;
+420 724 066 868

e-mail address of the corresponding author:

adam.eckhardt@fgu.cas.cz
