# Supplementary material for: Development of a 3D In Vitro Model of Dupuytren’s Disease as a Platform for Drug Screening
Source: Cell Mol Bioeng. 2026 Jan 19;19(1):111–27. doi: 10.1007/s12195-026-00885-2 (PMC13031596; doi:10.1007/s12195-026-00885-2)
Supplement: Supplementary file 3 — Comparison of standard and stepwise cultivations [file 12195_2026_885_MOESM3_ESM.pdf]

Additional file 3

Title: Comparison of standard and stepwise cultivation

The cells seeded on laminin-coated dECM cultivated in standard conditions, i.e., in DMEM medium with 10% FBS for 3 weeks, were compared to those that underwent stepwise cultivation with proliferation and differentiation phases as described in the Methods section. Stepwise cultivation led to deeper cell ingrowth and more even distribution of cells in the dECM. The immunofluorescence staining protocol corresponds to **Fig. 3A** in the manuscript.

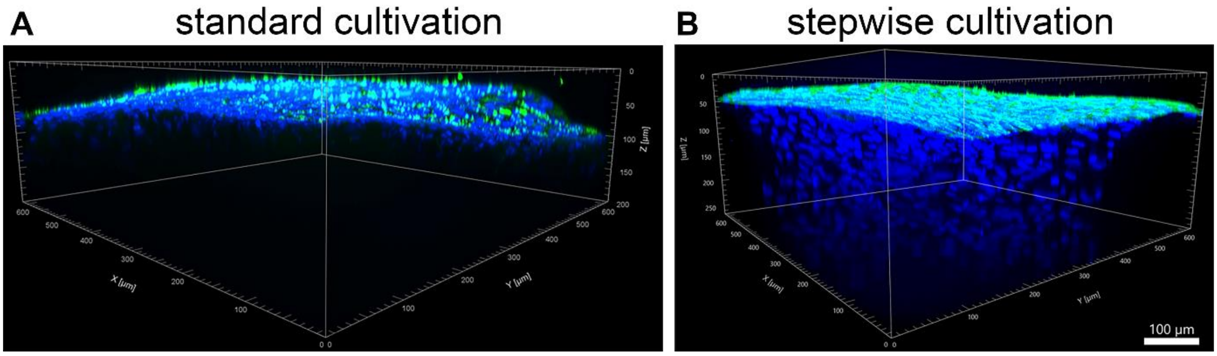

Fig. S1: **(A)** Cells cultivated in standard conditions, i.e., in DMEM medium with 10% FBS for 3 weeks. **(B)** Stepwise cultivation with proliferation (1 week) and differentiation (2 weeks) phases. Blue: nuclei, green: collagen type I.

Article title:

"Development of a 3D *in vitro* model of Dupuytren's Disease as a platform for drug screening"

Journal name:

Cellular and Molecular Bioengineering

Author names:

Jarmila Knitlova, Adam Eckhardt, Daniel Hadraba, David Vondrasek, Roman Stachon, Elena Filova, Vera Jencova, Kristyna Havlickova, Tatyana Kobets, Martin Ostadal and Lucie Bacakova

Affiliation:

Laboratory of Translational Metabolism,
Institute of Physiology of the Czech Academy of Sciences,
Videnska 1083, 142 00 Prague 4, Czech Republic;
+420 724 066 868

e-mail address of the corresponding author:

adam.eckhardt@fgu.cas.cz
