# Supplementary material for: Development of a 3D In Vitro Model of Dupuytren’s Disease as a Platform for Drug Screening
Source: Cell Mol Bioeng. 2026 Jan 19;19(1):111–27. doi: 10.1007/s12195-026-00885-2 (PMC13031596; doi:10.1007/s12195-026-00885-2)
Supplement: Supplementary file 4 — Proteomic data. PCA analysis and the list of significantly regulated proteins secreted by the cells cultivated on 3D dECM scaffolds [file 12195_2026_885_MOESM4_ESM.pdf]

Additional file 4

Title: Proteomic data

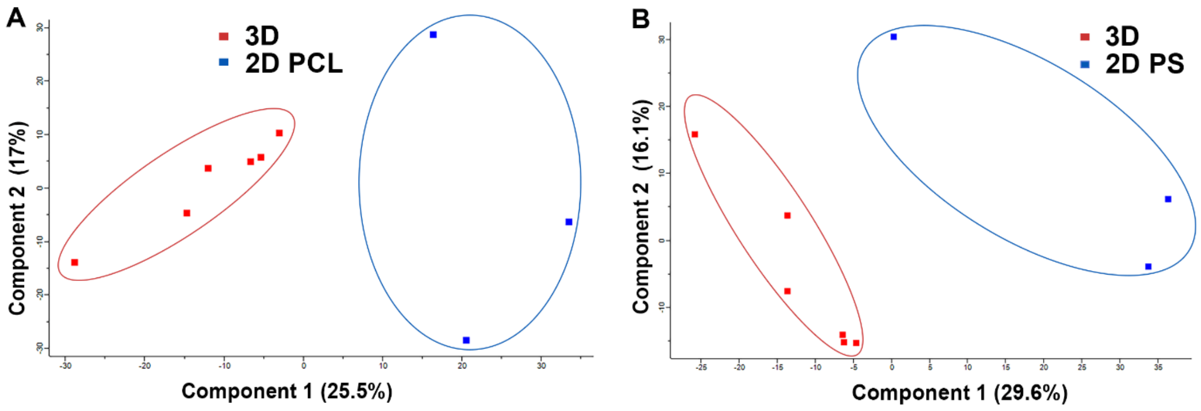

Fig. S1: Principal component analysis of proteomic data from 3D samples (left side, red) and 2D PCL (right side, blue) samples **(A)** and 3D (left side, red) and 2D PS (right side, blue) samples **(B)**.

Table S1: The list of significantly upregulated or downregulated proteins secreted by cells cultivated on 3D dECM scaffolds compared to 2D cultivation on a PCL nanomembrane **(3D x 2D PCL).** Positive difference values represents significantly upregulated proteins in 3D dECM scaffolds. Negative difference values represents significantly upregulated proteins in 2D PCL samples.

Difference (log2 fold)	-log10 P-value	Protein Descriptions	Genes
3.2397849	6.321686	Thrombospondin-4	THBS4
2.9244064	2.563371	Stromelysin-1	MMP3
2.6590279	2.740014	Collagen alpha-1(VII) chain	COL7A1
2.1210963	4.152033	Cartilage intermediate layer protein 1	CILP
2.0813068	2.364237	Interstitial collagenase	MMP1
2.0001823	2.721153	Tumor necrosis factor-inducible gene 6 protein	TNFAIP6
1.9079884	3.022317	Prelamin-A/C	LMNA
1.8636848	2.111287	Interleukin-6	IL6
1.6893708	3.055268	Heterogeneous nuclear ribonucleoproteins A2/B1	HNRNPA2B1
1.6788398	4.008009	Ribosome-binding protein 1	RRBP1
1.6696684	2.184736	Glucose-6-phosphate isomerase	GPI

1.6532365	3.526876	Vascular cell adhesion protein 1	VCAM1
1.6035638	2.955607	Glutathione S-transferase omega-1	GSTO1
1.6020737	1.67	Stanniocalcin-1	STC1
1.5534046	2.206551	Heterogeneous nuclear ribonucleoprotein K	HNRNPK
1.5082601	2.170277	Caldesmon	CALD1
1.4775008	2.43089	Collagen alpha-1(XI) chain	COL11A1
1.4229782	2.636318	Glutathione S-transferase P	GSTP1
1.4051312	2.627294	Large ribosomal subunit protein P2	RPLP2
1.4047111	2.565843	Chitinase-3-like protein 1	CHI3L1
1.3987773	3.201699	Agrin	AGRN
1.394214	2.159032	Collagen alpha-2(V) chain	COL5A2
1.3931045	1.826315	Small nuclear ribonucleoprotein Sm D3	SNRPD3
1.3922332	1.793244	Macrophage migration inhibitory factor	MIF
1.3432994	2.758259	Ferritin heavy chain	FTH1
1.3092494	2.695837	Procollagen-lysine.2-oxoglutarate 5-dioxygenase 2	PLOD2
1.2708826	3.462866	Collagen alpha-1(III) chain	COL3A1
1.2225785	2.407952	Protein S100-A9	S100A9
1.1888315	2.292451	Nicotinamide N-methyltransferase	NNMT
1.1645525	6.721154	Clusterin	CLU
1.1355699	2.724718	Plectin	PLEC
1.1162564	1.996502	Inhibin beta A chain	INHBA
1.0399027	1.993625	Fermitin family homolog 2	FERMT2
1.0217241	2.635685	Extracellular serine/threonine protein kinase FAM20C	FAM20C
1.012321	3.142812	Elongation factor 1-beta	EEF1B2
0.9854479	3.442984	Myosin regulatory light chain 12A and B	MYL12A
0.9755599	4.093027	Elongation factor 1-gamma	EEF1G
0.9699594	3.720523	Collagen type XVIII alpha 1 chain	COL18A1
0.9642502	3.941999	Latent-transforming growth factor beta-binding protein 1	LTBP1
0.9452799	2.330743	Angiopoietin-related protein 2	ANGPTL2
0.9423377	2.10381	Transforming growth factor beta-1 proprotein	TGFB1
0.9304578	2.173335	Triosephosphate isomerase	TPI1
0.9293094	2.286204	Fructose-bisphosphate aldolase A	ALDOA
0.9223304	2.045108	Myosin light polypeptide 6	MYL6
0.8856298	2.452357	EMILIN-1	EMILIN1
0.8707994	2.329626	Polypeptide N-acetylgalactosaminyltransferase 2	GALNT2
0.8232268	2.85967	Thioredoxin	TXN
0.7868179	2.084138	Procollagen lysine hydroxylase and glycosyltransferase LH3	PLOD3
0.7704722	2.333169	ADAMTS-like protein 1	ADAMTSL1
0.7261308	4.294124	Heat shock 70 kDa protein 1A	HSPA1A
0.6364109	2.30087	Tropomyosin alpha-4 chain	TPM4
0.6323646	2.695679	Tropomyosin beta chain	TPM2
0.5937994	3.927355	Filamin-A	FLNA
-0.520525	2.666561	Matrix-remodelling-associated protein 5	MXRA5
-0.6407007	2.489478	Laminin subunit alpha-2	LAMA2

-0.6581186	2.806448	Adenosylhomocysteinase	AHCY
-0.6923717	2.277662	Alpha-N-acetylgalactosaminidase	NAGA
-0.7605259	2.542709	Thyroglobulin	TG
-0.7837005	3.176184	Laminin subunit alpha-1	LAMA1
-0.7885319	2.061491	Alpha-fetoprotein	AFP
-0.7982798	1.994437	Pancreatic secretory granule membrane major glycoprotein	GP2
-0.811329	2.190867	Insulin-like growth factor-binding protein 7	IGFBP7
-0.8256736	2.609822	Vitamin K-dependent protein S	P07225
-0.8273637	2.24456	Sushi. von Willebrand factor type A. EGF and pentraxin domain-containing protein 1	SVEP1
-0.830236	2.178135	Microfibril-associated glycoprotein 4	MFAP4
-0.8359118	2.058646	Endosialin	CD248
-0.8506425	2.772816	Dihydropyrimidinase	DPYS
-0.9164713	2.002202	Neuronal cell adhesion molecule	NRCAM
-0.952258	2.637732	Alkaline phosphatase. tissue-nonspecific isozyme	ALPL
-0.9544964	2.323832	Spondin-2	SPON2
-0.9680622	2.521208	Inactive serine protease PAMR1	PAMR1
-0.9692942	1.881668	Fibrinogen beta chain	FGB
-0.9946136	2.309174	Alpha-1B-glycoprotein	A1BG
-1.0401673	1.980479	Golgi-associated plant pathogenesis-related protein 1	GLIPR2
-1.0470332	1.815318	Lactadherin	MFGE8
-1.077271	2.711003	Lysosome-associated membrane glycoprotein 1	LAMP1
-1.0840815	1.965369	Cytokine receptor-like factor 1	CRLF1
-1.1102363	2.337991	Hemoglobin subunit alpha	HBA1
-1.1205926	4.973148	Dipeptidyl peptidase 4	DPP4
-1.1801651	2.490791	Fibulin-2	FBLN2
-1.2260106	2.107291	Plakophilin-1	PKP1
-1.3557487	2.207372	DBH-like monooxygenase protein 1	MOXD1
-1.4152617	2.138281	Desmoglein-2	DSG2
-1.4499216	4.415541	Galectin-3-binding protein	LGALS3BP
-1.4981817	2.301547	Retinoic acid receptor responder protein 2	RARRES2
-1.4988872	1.993001	Carboxypeptidase E	CPE
-1.5454685	2.897685	2-iminobutanoate/2-iminopropanoate deaminase	RIDA
-1.5770139	2.426815	Fibrillin-2	FBN2
-1.5926886	2.883293	Transmembrane glycoprotein NMB	GPNMB
-1.6249299	2.078428	Ciliary neurotrophic factor receptor subunit alpha	CNTFR
-1.7555122	1.636981	Acid ceramidase	ASAH1
-1.8262968	1.895955	Dickkopf-related protein 1	DKK1
-1.9008668	2.120507	Serum amyloid P-component	APCS
-1.9397957	4.020743	Peptidase inhibitor 16	PI16
-1.9480225	2.609845	Collagen alpha-1(XV) chain	COL15A1
-1.9490698	3.492409	Testis-specific serine/threonine-protein kinase 4	TSSK4
-2.0248251	1.957457	N-acetylglucosamine-6-sulfatase	GNS
-2.1616162	2.641015	CCN family member 5	CCN5
-2.1734028	2.190038	Prostaglandin-H2 D-isomerase	PTGDS

-2.6321354	2.385997	Podocan	PODN

Table S2: The list of significantly upregulated or downregulated proteins secreted by cells cultivated on 3D dECM scaffolds compared to 2D cultivation on a polystyrene well-plate (**3D x 2D PS**). Positive difference values represents significantly upregulated proteins in 3D dECM scaffolds. Negative difference values represents significantly upregulated proteins in 2D PS samples.

Difference (log2 fold)	-log10 P-value	Protein Descriptions	Genes
3.5890077	6.618519	Thrombospondin-4	THBS4
2.9579042	2.884842	HLA class I histocompatibility antigen. B alpha chain	HLA-B
2.8185271	5.812563	Collagen alpha-1(VII) chain	COL7A1
2.3809799	3.580074	Hyaluronan and proteoglycan link protein 1	HAPLN1
2.2940415	4.80094	Vascular cell adhesion protein 1	VCAM1
2.2865898	3.295877	CCN family member 1	CCN1
2.2500045	2.488147	Stromelysin-1	MMP3
2.1789252	2.350116	Interstitial collagenase	MMP1
2.0845443	2.163039	Interleukin-6	IL6
2.0795121	5.093477	Collagen alpha-1(XI) chain	COL11A1
2.0142409	4.622644	Agrin	AGRN
1.9951968	2.779397	Collagen alpha-2(IV) chain	COL4A2
1.967848	3.327221	Glutathione S-transferase omega-1	GSTO1
1.9611111	6.489386	Collagen alpha-2(V) chain	COL5A2
1.8849297	5.881568	Matrix remodelling-associated protein 8	MXRA8
1.8815792	1.589992	Macrophage migration inhibitory factor	MIF
1.869933	1.943449	Collagen alpha-1(IV) chain	COL4A1
1.8228555	2.601239	Tumor necrosis factor-inducible gene 6 protein	TNFAIP6
1.802039	1.94335	Gremlin-1	GREM1
1.7917614	2.806842	Cartilage intermediate layer protein 1	CILP
1.7457242	1.85657	Ribosome-binding protein 1	RRBP1
1.7397389	4.104458	Clusterin	CLU
1.6996034	2.161631	Platelet-derived growth factor receptor-like protein	PDGFRL
1.6599704	2.653775	Prelamin-A/C	LMNA
1.6452314	6.191994	Anthrax toxin receptor 1	ANTXR1
1.6368655	4.571594	Angiopoietin-related protein 2	ANGPTL2
1.6264923	3.754458	Procollagen-lysine.2-oxoglutarate 5-dioxygenase 2	PLOD2
1.5630935	2.909969	Chitinase-3-like protein 1	CHI3L1
1.5459027	1.579992	Cartilage intermediate layer protein 2	CILP2

1.5267909	5.779448	Procollagen-lysine.2-oxoglutarate 5-dioxygenase 1	PLOD1
1.5037165	2.697441	Matrix metalloproteinase-14	MMP14
1.4430081	2.345645	Phosphoacetylglucosamine mutase	PGM3
1.4189013	1.597016	Protein Wnt-5a	WNT5A
1.4154582	4.304454	Disintegrin and metalloproteinase domain-containing protein 9	ADAM9
1.4003105	5.415669	Collagen alpha-1(V) chain	COL5A1
1.3775392	2.007694	Glucose-6-phosphate isomerase	GPI
1.3723421	4.711301	Latent-transforming growth factor beta-binding protein 1	LTBP1
1.3548191	3.858053	Olfactomedin-like protein 2B	OLFML2B
1.3197322	2.596416	Glutathionse S-transferase P	GSTP1
1.2911266	3.169119	Periostin	POSTN
1.277516	2.033809	ADAMTS-like protein 1	ADAMTSL1
1.2751746	1.607965	Myoferlin	MYOF
1.2134693	4.135639	Amyloid-beta precursor protein	APP
1.1992213	2.483743	Urokinase-type plasminogen activator	PLAU
1.1913039	2.028093	Nucleobindin-1	NUCB1
1.1846981	3.266821	Dickkopf-related protein 3	DKK3
1.1822901	1.683727	Ubiquitin-conjugating enzyme E2 N	UBE2N
1.1701299	4.37392	Coiled-coil domain-containing protein 80	CCDC80
1.1469676	3.522348	Interleukin enhancer-binding factor 2	ILF2
1.1402941	3.417191	Fibronectin type III domain-containing protein 1	FNDC1
1.1318789	2.709508	CCN family member 2	CCN2
1.1186288	2.091204	Triosephosphate isomerase	TPI1
1.1038074	1.873364	Inhibin beta A chain	INHBA
1.0879812	2.435397	SPARC	SPARC
1.084764	4.455552	Polypeptide N-acetylgalactosaminyltransferase 5	GALNT5
1.0819747	3.290254	Transforming growth factor beta-1 proprotein	TGFB1
1.0781864	2.894562	Exostosin-2	EXT2
1.0741612	1.743383	Heterogeneous nuclear ribonucleoprotein A1	HNRNPA1
1.065969	1.917094	Plectin	PLEC
1.053966	2.348999	Polypeptide N-acetylgalactosaminyltransferase 10	GALNT10
1.0446952	3.475895	Collagen alpha-1(III) chain	COL3A1
1.0318413	4.403116	Extracellular sulfatase Sulf-2	SULF2
1.0222108	2.009021	Peroxiredoxin-4	PRDX4
1.0190001	2.469652	Follistatin-related protein 1	FSTL1
0.9915435	3.0719	Vesicular integral-membrane protein VIP36	LMAN2
0.984062	2.68214	CD276 antigen	CD276
0.975958	2.341909	Ferritin heavy chain	FTH1
0.9726504	2.307926	Soluble scavenger receptor cysteine-rich domain-containing protein SSC5D	SSC5D
0.9697212	1.726107	Ras GTPase-activating-like protein IQGAP1	IQGAP1
0.9651833	5.025164	Collagen alpha-3(VI) chain	COL6A3
0.9624917	2.058074	Ribonuclease T2	RNASET2
0.9579206	3.099083	Beta-mannosidase	MANBA
0.952287	2.952761	Endoplasmic reticulum aminopeptidase 1	ERAP1

0.9386237	2.409338	Transaldolase	TALDO1
0.9348879	1.995565	Extracellular serine/threonine protein kinase FAM20C	FAM20C
0.9309276	3.41896	Collagen triple helix repeat-containing protein 1	CTHRC1
0.9066922	6.004931	Multifunctional procollagen lysine hydroxylase and glycosyltransferase LH3	PLOD3
0.8959036	2.062425	Prolyl 4-hydroxylase subunit alpha-2	P4HA2
0.8826947	4.403065	Procollagen C-endopeptidase enhancer 1	PCOLCE
0.8510119	3.06646	Collectin-12	COLEC12
0.8180896	3.479349	Collagen type XVIII alpha 1 chain	COL18A1
0.8081641	2.100706	Cathepsin F	CTSF
0.8040627	3.080181	Galectin-1	LGALS1
0.800018	3.47666	Fibronectin	FN1
0.7976281	4.317479	C-type mannose receptor 2	MRC2
0.7863647	2.316926	Polypeptide N-acetylgalactosaminyltransferase 2	GALNT2
0.7839675	2.508207	Filamin-B	FLNB
0.7804993	2.901312	Cathepsin Z	CTSZ
0.7705177	2.337907	Mannosyl-oligosaccharide 1.2-alpha-mannosidase IA	MAN1A1
0.7597087	2.352179	Isocitrate dehydrogenase [NADP] cytoplasmic	IDH1
0.7572333	1.995012	Complement C1r subcomponent-like protein	C1RL
0.7492402	1.938883	Proteasome subunit alpha type-5	PSMA5
0.7487473	2.078351	Nicotinamide N-methyltransferase	NNMT
0.7348948	1.974703	Lysosomal alpha-glucosidase	GAA
0.733333	4.627352	C-type lectin domain family 11 member A	CLEC11A
0.6975867	2.582231	Complement C3	C3
0.6942306	2.305217	Dystroglycan 1	DAG1
0.6195641	2.668567	Collagen alpha-2(VI) chain	COL6A2
0.61261	2.944034	Complement factor H	CFH
0.6088503	3.060636	Chondroitin sulfate proteoglycan 4	CSPG4
0.5993028	2.985079	Extracellular matrix protein 1	ECM1
0.5918059	2.212916	Lysyl oxidase homolog 2	LOXL2
0.4649463	2.703602	Collagen alpha-1(XVI) chain	COL16A1
0.4308124	2.989973	Peroxidasin homolog	PXDN
0.3944276	3.717538	Gelsolin	GSN
-0.597483	3.053493	Matrix-remodeling-associated protein 5	MXRA5
-0.6002221	3.623027	Laminin subunit alpha-1	LAMA1
-0.6247362	2.547677	Adenosylhomocysteinase	AHCY
-0.6295215	2.701397	Glycogen phosphorylase. liver form	PYGL
-0.6455371	2.24133	N-acetylglucosamine-6-sulfatase	GNS
-0.6613102	2.407935	Argininosuccinate synthase	ASS1
-0.6896871	3.291756	Collagen alpha-1(VIII) chain	COL8A1
-0.7955669	2.40986	Mimecan	OGN
-0.799156	1.867186	Cathepsin D	CTSD
-0.823657	2.207747	Sushi. von Willebrand factor type A. EGF and pentraxin domain-containing protein 1	SVEP1
-0.8254251	1.969027	Insulin-like growth factor 2	IGF2
-0.8568147	2.637265	Collagen alpha-1(I) chain	COL1A1
-0.8836784	2.384947	Desmoglein-2	DSG2

-0.8906639	2.587507	Insulin-like growth factor-binding protein 7	IGFBP7
-0.9564731	1.809026	Leukocyte cell-derived chemotaxin-2	LECT2
-0.964979	2.944078	Dipeptidyl peptidase 4	DPP4
-1.0102959	3.282434	Platelet-derived growth factor D	PDGFD
-1.0116541	2.404969	Multiple epidermal growth factor-like domains protein 6	MEGF6
-1.017415	1.860456	Transmembrane glycoprotein NMB	GPNMB
-1.0368991	4.182657	Inactive serine protease PAMR1	PAMR1
-1.0555198	2.034221	Rho GTPase-activating protein 1	ARHGAP1
-1.0588981	2.148292	Complement factor D	CFD
-1.0959444	3.893132	Galectin-3-binding protein	LGALS3BP
-1.2721949	2.532108	Stanniocalcin-2	STC2
-1.2835815	2.809365	DnaJ homolog subfamily B member 11	DNAJB11
-1.2884084	2.522839	Sushi repeat-containing protein SRPX2	SRPX2
-1.2993108	1.60699	EMILIN-3	EMILIN3
-1.4129517	2.136831	Keratin. type I cytoskeletal 19	KRT19
-1.4974516	3.721739	Fibulin-2	FBLN2
-1.5686851	2.532919	Phosphoserine aminotransferase	PSAT1
-1.5967748	1.660222	Olfactomedin-like protein 1	OLFML1
-1.6349168	2.866409	Testis-specific serine/threonine-protein kinase 4	TSSK4
-1.7053528	3.026184	Retinoic acid receptor responder protein 2	RARRES2
-1.744545	1.746416	Catalase	CAT
-1.8164666	1.635032	Trypsin-3	PRSS3
-1.8782738	3.529364	CCN family member 5	CCN5
-1.890039	3.840999	Peptidase inhibitor 16	PI16
-1.9228479	1.908322	Hemoglobin subunit gamma-1	HBE1
-1.935856	2.418885	2-iminobutanoate/2-iminopropanoate deaminase	RIDA
-1.9370879	2.812303	Carboxypeptidase Z	CPZ
-1.9458672	4.596191	Pappalysin-1	PAPPA
-2.16914	2.41229	Cytokine receptor-like factor 1	CRLF1
-2.4529778	3.376044	Pentraxin-related protein PTX3	PTX3
-2.5372396	1.430599	Junction plakoglobin	JUP
-2.5666676	1.709781	Prostaglandin-H2 D-isomerase	PTGDS
-2.717631	1.585134	Desmocollin-3	DSC3
-2.8422916	3.049425	Plakophilin-1	PKP1
-2.9975125	5.042202	Collagen alpha-1(XV) chain	COL15A1
-3.1158489	1.386091	Keratin, type I cytoskeletal 9	KRT9
-3.344718	2.883219	Podocan	PODN
-3.8822007	2.198358	Keratin, type II cytoskeletal 4	KRT4

Article title:

“Development of a 3D *in vitro* model of Dupuytren’s Disease as a platform for drug screening”

Journal name:

Cellular and Molecular Bioengineering

Author names:

Jarmila Knitlova, Adam Eckhardt, Daniel Hadraba, David Vondrasek, Roman Stachon, Elena Filova, Vera Jencova, Kristyna Havlickova, Tatyana Kobets, Martin Ostadal and Lucie Bacakova

Affiliation:

Laboratory of Translational Metabolism,
Institute of Physiology of the Czech Academy of Sciences,
Videnska 1083, 142 00 Prague 4, Czech Republic;
+420 724 066 868

e-mail address of the corresponding author:

adam.eckhardt@fgu.cas.cz
