# Supplementary material for: Development of a 3D In Vitro Model of Dupuytren’s Disease as a Platform for Drug Screening
Source: Cell Mol Bioeng. 2026 Jan 19;19(1):111–27. doi: 10.1007/s12195-026-00885-2 (PMC13031596; doi:10.1007/s12195-026-00885-2)
Supplement: Supplementary file 5 — Proteomic data. PCA analysis of secretomes of control and minoxidil treated 3D samples. The list of significantly regulated proteins secreted by the cells cultivated on 3D dECM scaffolds with/without minoxidil [file 12195_2026_885_MOESM5_ESM.pdf]

Additional file 5

Title: Proteomic analysis of secretomes of control and minoxidil-treated 3D samples

Fig.S1: The Volcano plot shows upregulated (red, right side) and downregulated (blue, left side) proteins in 3D control samples compared to 3D MXD-treated samples (n=3).

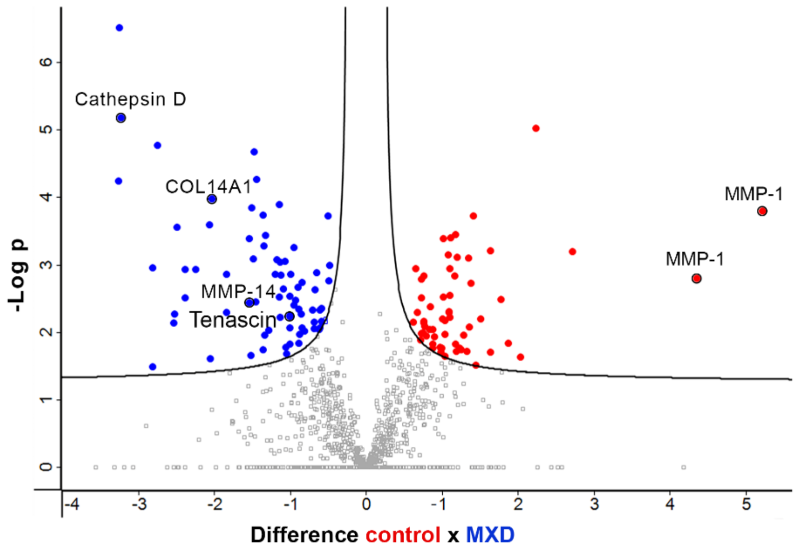

This figure demonstrates that significant effect of minoxidil is detectable on the 3D model even with a sample size of only n=3.

Fig.S2: Principal component analysis shows two distinguishable clusters of 3D control cells (left side, red) and minoxidil-treated cells (right side, blue)**.**

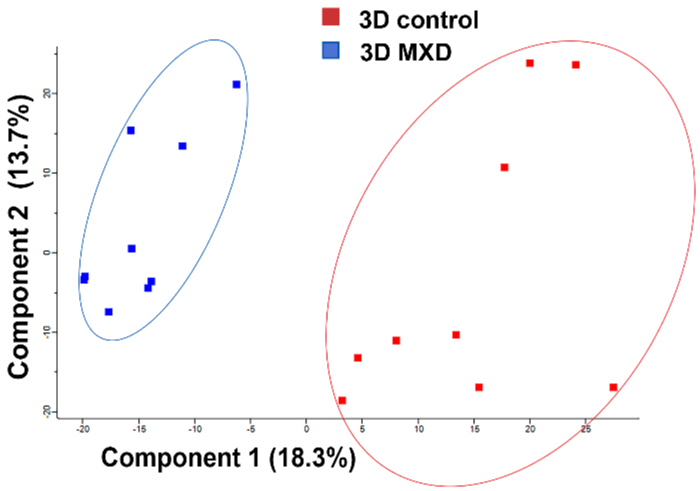

Table S1: The list of significantly regulated proteins secreted by cells cultivated on 3D dECM scaffolds compared to minoxidil-treated 3D samples (**3D control x 3D MXD**). Positive difference values represents significantly upregulated proteins in 3D control samples, negative difference values represents significantly downregulated proteins in 3D control samples.

Difference (Log2 fold)	-log10 P-value	Protein Descriptions	Genes
4.2006666	5.972285	Stromelysin-1	MMP3
3.6860037	4.183011	Interstitial collagenase	MMP1
2.3225639	2.250837	Interleukin-6	IL6
2.1048436	4.739188	Protein NDNF	NDNF
1.931937	2.263827	Hyaluronan and proteoglycan link protein 1	HAPLN1
1.7592255	2.820954	Angiopoietin-1	ANGPT1
1.6355241	3.050368	Dihydropyrimidinase-related protein 3	DPYSL3
1.5720601	2.285096	Collagenase 3	MMP13
1.4567024	2.129049	Stanniocalcin-2	STC2
1.4501949	4.085386	Insulin-like growth factor-binding protein 4	IGFBP4
1.4322306	1.995988	Collagen alpha-1(VII) chain	COL7A1
1.3937175	2.6882	Tubulin-specific chaperone A	TBCA
1.3823536	1.795829	Septin-11	SEPTIN11
1.3812029	2.700843	Perilipin-3	PLIN3
1.3804957	4.969661	Interleukin enhancer-binding factor 2	ILF2
1.3009046	3.378245	Importin-5	IPO5
1.2640974	4.342889	Neutral alpha-glucosidase AB	GANAB
1.2425392	3.061737	Caldesmon	CALD1
1.2314178	3.890692	Coronin-1C	CORO1C
1.2279973	2.909792	Tropomyosin alpha-1 chain	TPM1
1.1890268	1.609243	Protein Wnt-5a	WNT5A
1.1716795	3.509451	ADP-ribosylation factor 4	ARF4
1.1712955	3.480682	Actin-related protein 2	ACTR2
1.1653821	2.237964	MAM domain-containing protein 2	MAMDC2

1.1549065	4.092141	Nucleolin	NCL
1.1360879	7.050992	Fructose-bisphosphate aldolase A	ALDOA
1.1337682	1.683565	Heterogeneous nuclear ribonucleoprotein A/B	HNRNPAB
1.1273724	6.420156	Parathymosin	PTMS
1.1256581	2.891969	Translationally-controlled tumor protein	TPT1
1.1061218	3.832733	Tryptophan--tRNA ligase. cytoplasmic	WARS1
1.105343	1.826731	Gremlin-1	GREM1
1.0999083	2.07251	Ferritin heavy chain	FTH1
1.0939887	2.014682	Nuclear ubiquitous casein and cyclin-dependent kinase substrate 1	NUCKS1
1.0928104	1.719664	Microtubule-associated protein RP/EB family member 1	MAPRE1
1.0927574	4.492721	Glia-derived nexin	SERPINE2
1.0399107	1.831622	Inhibin beta A chain	INHBA
1.0351521	1.636477	Tumor necrosis factor-inducible gene 6 protein	TNFAIP6
1.0130826	3.303527	Decorin	DCN
0.9990553	2.81295	Plasminogen activator inhibitor 1	SERPINE1
0.9989973	4.731957	L-lactate dehydrogenase A chain	LDHA
0.9908758	2.176164	Tumor protein D54	TPD52L2
0.9868168	1.858866	Fatty acid synthase	FASN
0.9842823	3.421444	Phosphoacetylglucosamine mutase	PGM3
0.9748727	1.799564	Prolyl 4-hydroxylase subunit alpha-1	P4HA1
0.9652163	4.098956	Ribosome-binding protein 1	RRBP1
0.9504375	3.083549	Actin-related protein 2/3 complex subunit 3	ARPC3
0.9501044	2.131679	Calnexin	CANX
0.9475889	4.13595	Large ribosomal subunit protein uL1	RPL10A
0.9371297	3.887873	Glucosidase 2 subunit beta	PRKCSH
0.9245264	2.53472	Transgelin	TAGLN
0.9238682	3.584421	Follistatin	FST
0.9227428	5.791218	Myosin regulatory light chain 12A	MYL12A
0.9226625	2.991067	Plectin	PLEC
0.9182766	2.774097	Peptidyl-prolyl cis-trans isomerase B	PPIB
0.9161622	2.525369	Metalloproteinase inhibitor 3	TIMP3
0.9141983	3.855295	Large ribosomal subunit protein P2	RPLP2
0.8956932	4.320632	ATP synthase subunit alpha. mitochondrial	ATP5F1A
0.8933935	4.183282	Follistatin-related protein 1	FSTL1
0.8871497	3.308506	Vimentin	VIM
0.8693404	3.345272	Macrophage migration inhibitory factor	MIF
0.8377437	6.54258	UTP--glucose-1-phosphate uridylyltransferase	UGP2
0.8371332	3.444733	Phosphoglycerate kinase 1	PGK1
0.8304584	4.653764	CCN family member 1	CCN1
0.8303065	2.851914	Hepatoma-derived growth factor	HDGF
0.8257909	3.885122	Elongation factor 1-beta	EEF1B2
0.8189763	4.520139	Fascin	FSCN1
0.8061534	2.430172	Xylosyltransferase 1	XYLT1
0.8042788	3.121894	Filamin-C	FLNC
0.7963723	3.647104	Nicotinamide N-methyltransferase	NNMT
0.793296	1.981764	Elongin-B	ELOB
0.7929833	4.735622	Endoplasmic reticulum resident protein 44	ERP44
0.7687134	1.772244	Prelamin-A/C	LMNA
0.7574423	5.110822	Myosin light polypeptide 6	MYL6

0.754856	3.442939	Coatomer subunit beta'	COPB2
0.7544617	2.855632	Large ribosomal subunit protein uL10	RPLP0
0.75405	2.043815	Astrocytic phosphoprotein PEA-15	PEA15
0.7483661	2.578188	Macrophage colony-stimulating factor 1	CSF1
0.7448743	2.694426	Thioredoxin domain-containing protein 5	TXNDC5
0.7428646	1.853482	Actin-related protein 2/3 complex subunit 1B	ARPC1B
0.7414224	6.395767	Protein disulfide-isomerase	P4HB
0.7384295	1.888006	Tubulin beta-3 chain	TUBB3
0.7382547	2.968494	Protein disulfide-isomerase A4	PDIA4
0.7379394	2.701204	Heat shock 70 kDa protein 1A	HSPA1A
0.7334642	2.762556	Protein disulfide-isomerase A6	PDIA6
0.7317074	1.860918	Small ribosomal subunit protein RACK1	RACK1
0.7153225	2.376765	Spectrin alpha chain. non-erythrocytic 1	SPTAN1
0.7042909	2.511319	Small ribosomal subunit protein eS1	RPS3A
0.7031431	3.866791	ATP synthase subunit beta. mitochondrial	ATP5F1B
0.7021092	1.983744	Heat shock 70 kDa protein 4	HSPA4
0.698383	3.931596	Glypican-1	GPC1
0.6931825	4.415819	Elongation factor 1-gamma	EEF1G
0.6924553	2.966593	Translin	TSN
0.6883297	2.638843	Protein disulfide-isomerase A3	PDIA3
0.679974	3.618374	Filamin-B	FLNB
0.6786207	1.966186	Bifunctional phosphoribosylaminoimidazole carboxylase	PAICS
0.6740425	2.587336	U6 snRNA-associated Sm-like protein LSm3	LSM3
0.6685412	2.208348	Insulin-like growth factor-binding protein 6	IGFBP6
0.663128	2.059153	Nuclear migration protein nudC	NUDC
0.662154	3.620546	Actin-related protein 3	ACTR3
0.6583123	1.867711	Inactive serine protease PAMR1	PAMR1
0.6374131	2.177889	Heterogeneous nuclear ribonucleoprotein A1-like 3	HNRNPA1L3
0.6368725	2.625874	Heterogeneous nuclear ribonucleoproteins A2/B1	HNRNPA2B1
0.6339142	2.89924	F-actin-capping protein subunit alpha-2	CAPZA2
0.6272019	1.892163	Serpin H1	SERPINH1
0.6242149	3.026924	Growth arrest-specific protein 6	GAS6
0.6218704	3.164111	Ubiquitin thioesterase OTUB1	OTUB1
0.6211673	2.919071	Alpha-enolase	ENO1
0.6180485	2.138213	Protein TFG	TFG
0.6164618	2.226457	Alpha-1.3-mannosyl-glycoprotein 2-beta-N-acetylglucosaminyltransferase	MGAT1
0.6033681	4.069432	Alpha-actinin-4	ACTN4
0.5976133	2.409737	14-3-3 protein eta	YWHAH
0.594329	2.909502	Small nuclear ribonucleoprotein E	SNRPE
0.5937625	3.771994	Tropomyosin beta chain	TPM2
0.5914818	2.629114	Annexin A2	ANXA2
0.5909925	5.195402	Galectin-1	LGALS1
0.588194	4.722443	Transitional endoplasmic reticulum ATPase	VCP
0.5853196	3.694765	Hsc70-interacting protein	ST13
0.5740468	2.94165	Ubiquitin carboxyl-terminal hydrolase isozyme L1	UCHL1
0.5543684	2.273784	Endoplasmin	HSP90B1
0.550236	2.560253	Complement C3	C3
0.5431726	5.984168	S-phase kinase-associated protein 1	SKP1

0.5424964	2.746153	Vasorin	VASN
0.5391171	2.537353	Ribonuclease inhibitor	RNH1
0.5357399	4.785979	Alpha-actinin-1	ACTN1
0.5217874	2.73017	Triosephosphate isomerase	TPI1
0.5169095	2.282776	EGF-containing fibulin-like extracellular matrix protein 1	EFEMP1
0.5142701	2.46057	GTP-binding nuclear protein Ran	RAN
0.49964	4.448001	Filamin-A	FLNA
0.4967661	2.411355	WD repeat-containing protein 1	WDR1
0.4865253	4.225641	Actin-related protein 2/3 complex subunit 4	ARPC4
0.4724316	2.824736	Elongation factor 2	EEF2
0.4706434	2.940062	Spliceosome RNA helicase DDX39B	DDX39B
0.454479	2.479444	Nucleoside diphosphate kinase B	NME2
0.4208784	2.973574	14-3-3 protein zeta/delta	YWHAZ
0.3901133	3.109373	Heat shock protein HSP 90-beta	HSP90AB1
-0.4032129	2.717572	A disintegrin and metalloproteinase with thrombospondin motifs 13	ADAMTS13
-0.4225603	4.255165	Neogenin	NEO1
-0.4524974	2.771265	Plasminogen	PLG
-0.4559956	2.721808	Platelet-activating factor acetylhydrolase	PLA2G7
-0.4583182	2.628008	Inactive tyrosine-protein kinase 7	PTK7
-0.4652591	2.22129	Cis-aconitate decarboxylase	ACOD1
-0.4754601	2.463951	Adipocyte enhancer-binding protein 1	AEBP1
-0.4801727	3.869261	Plasma protease C1 inhibitor	SERPING1
-0.4898273	3.266842	Reelin	RELN
-0.4920965	3.598095	Plexin-B2	PLXNB2
-0.5073449	2.566156	Collagen alpha-1(IX) chain	COL9A1
-0.5073696	2.932389	Putative hydrolase DDAH2	DDAH2
-0.5108878	4.22872	Protocadherin gamma-C3	PCDHGC3
-0.511037	4.288139	C-type mannose receptor 2	MRC2
-0.5187088	6.563988	Prolow-density lipoprotein receptor-related protein 1	LRP1
-0.5295773	2.476191	Testican-1	SPOCK1
-0.5525769	5.053807	Fibrocystin-L	PKHD1L1
-0.5699447	2.463704	Cathepsin B	CTSB
-0.5719637	2.755357	Apolipoprotein B-100	APOB
-0.5733508	3.690367	Mannan-binding lectin serine protease 2	MASP2
-0.5813276	3.363383	N-acetylglucosamine-1-phosphotransferase subunit gamma	GNPTG
-0.5887616	2.881296	Lactadherin	MFGE8
-0.6014158	2.189224	Vitronectin	VTN
-0.614691	2.317336	Disintegrin and metalloproteinase domain-containing protein 12	ADAM12
-0.6295904	1.929368	Beta-2-microglobulin	B2M
-0.6332126	3.018705	Complement C4-A	C4A
-0.6415688	2.912655	Transferrin receptor protein 1	TFRC
-0.6482927	2.98749	Adipocyte plasma membrane-associated protein	APMAP
-0.6592384	2.756384	Receptor-type tyrosine-protein phosphatase S	PTPRS
-0.6836651	2.24282	Lysosomal alpha-glucosidase	GAA
-0.7143846	1.818537	Immunoglobulin superfamily containing leucine-rich repeat protein	ISLR
-0.7164255	6.281596	Thrombospondin-2	THBS2
-0.7187485	3.267091	Collagen alpha-1(VIII) chain	COL8A1

-0.7211651	3.06988	Amyloid-beta precursor protein	APP
-0.7303313	1.694831	Zinc transporter ZIP10	SLC39A10
-0.731005	1.744369	Beta-mannosidase	MANBA
-0.7439605	1.829765	Fibrinogen gamma chain	FGG
-0.7604418	3.89794	Alpha-N-acetylgalactosaminidase	NAGA
-0.7844403	1.820962	Centrosomal protein of 44 kDa	CEP44
-0.7959378	2.299057	Matrix remodeling-associated protein 8	MXRA8
-0.8072019	4.012672	Alpha-mannosidase 2	MAN2A1
-0.821928	2.80124	Trans-Golgi network integral membrane protein 2	TGOLN2
-0.8220376	3.399029	Plexin-A1	PLXNA1
-0.8307063	5.546434	Vitamin K-dependent protein S	XII.01
-0.8420398	2.612387	HLA class I histocompatibility antigen. C alpha chain	HLA-C
-0.8488203	4.335089	Collectin-12	COLEC12
-0.8490216	4.469423	Carboxypeptidase D	CPD
-0.8626509	1.751181	CCN family member 5	CCN5
-0.8900201	6.458173	Neuropilin-2	NRP2
-0.9063637	7.57258	CD109 antigen	CD109
-0.9118953	5.629823	Plexin domain-containing protein 2	PLXDC2
-0.9130312	2.083759	Ganglioside GM2 activator	GM2A
-0.9245368	1.973254	Catalase	CAT
-0.9512811	4.995283	Bis(monoacylglycero)phosphate synthase CLN5	CLN5
-0.9821974	5.046476	Lysosomal Pro-X carboxypeptidase	PRCP
-0.9827993	2.608842	Amino acid transporter heavy chain SLC3A2	SLC3A2
-0.9836912	2.245518	CD63 antigen	CD63
-0.9984239	6.202878	Golgi membrane protein 1	GOLM1
-1.01425	1.927179	Bleomycin hydrolase	BLMH
-1.0280153	4.15756	Alpha-N-acetylglucosaminidase	NAGLU
-1.0327902	2.404252	Platelet-derived growth factor receptor alpha	PDGFRA
-1.0351339	5.07799	Lysosomal alpha-mannosidase	MAN2B1
-1.0376594	3.468988	Arylsulfatase B	ARSB
-1.0406704	2.26289	Endoglin	ENG
-1.0494186	2.155887	Desmoglein-2	DSG2
-1.0836291	6.313069	Complement C2	C2
-1.1158131	4.677109	CD276 antigen	CD276
-1.117074	4.56906	Phospholipid transfer protein	PLTP
-1.1369237	4.379726	Heat shock 70 kDa protein 13	HSPA13
-1.1381276	1.617934	Probable carboxypeptidase X1	CPXM1
-1.1823028	3.049037	Dipeptidyl peptidase 1	CTSC
-1.190018	5.114923	Cathepsin Z	CTSZ
-1.1959047	5.781841	Ribonuclease T2	RNASET2
-1.2294402	4.949275	Tenascin	TNC
-1.2316857	1.65723	General transcription factor IIH subunit 1	GTF2H1
-1.2619845	7.630517	Prostaglandin F2 receptor negative regulator	PTGFRN
-1.2627356	4.762917	Procathepsin L	CTSL
-1.2701636	6.436894	Tissue alpha-L-fucosidase	FUCA1
-1.2710819	8.794502	Beta-hexosaminidase subunit alpha	HEXA
-1.2733686	9.807522	Olfactomedin-like protein 2B	OLFML2B
-1.3142324	3.085413	Cathepsin F	CTSF
-1.3432242	5.501765	Spondin-2	SPON2
-1.3813071	8.755637	Platelet-derived growth factor receptor beta	PDGFRB

-1.405719	1.638026	Prolargin	PRELP
-1.4309013	5.40614	Carboxypeptidase Q	CPQ
-1.4412749	4.616171	Matrix metalloproteinase-14	MMP14
-1.4458578	3.373006	Leucyl-cystinyl aminopeptidase	LNPEP
-1.454384	3.766354	Dipeptidyl peptidase 2	DPP7
-1.5397497	2.979579	Neuroserpin	SERPINI1
-1.5547797	6.162131	Progranulin	GRN
-1.5699619	3.882256	Arylsulfatase A	ARSA
-1.6142628	8.984428	Sphingomyelin phosphodiesterase	SMPD1
-1.7129185	1.963898	Stomatin	STOM
-1.7879157	2.810917	N-sulphoglucosamine sulphohydrolase	SGSH
-1.8455135	7.229758	Mammalian ependymin-related protein 1	EPDR1
-1.8714223	11.08257	Beta-hexosaminidase subunit beta	HEXB
-1.9045227	1.856671	Cathepsin K	CTSK
-1.9093432	7.771322	Acid ceramidase	ASAH1
-1.911202	2.824549	Keratin. type II cytoskeletal 4	KRT4
-1.9948122	3.898811	Beta-1.4-galactosyltransferase 5	B4GALT5
-1.9990543	7.495432	Complement C1q tumor necrosis factor-related protein 5	C1QTNF5
-2.0866733	10.23743	Putative phospholipase B-like 2	PLBD2
-2.1132725	4.104261	Collagen alpha-1(XIV) chain	COL14A1
-2.1213402	3.239246	Stromelysin-3	MMP11
-2.1662671	3.901655	Transmembrane glycoprotein NMB	GPNMB
-2.2847521	1.567911	Neuroendocrine convertase 1	PCSK1
-2.3422839	6.650033	Epididymis-specific alpha-mannosidase	MAN2B2
-2.4180187	3.568504	Protein CREG1	CREG1
-2.4975033	4.633193	Lysosomal acid lipase/cholesteryl ester hydrolase	LIPA
-2.555648	5.200663	Legumain	LGMN
-2.5769937	7.926305	Deoxyribonuclease-2-alpha	DNASE2
-2.7287503	6.644318	Cyclic GMP-AMP phosphodiesterase SMPDL3A	SMPDL3A
-2.9121206	12.10279	Tripeptidyl-peptidase 1	TPP1
-2.9351815	4.241702	Sialidase-1	NEU1
-2.9749832	12.49125	Cathepsin D	CTSD
-2.9864431	12.06962	Prosaposin	PSAP
-3.0439835	10.65978	N-acetylglucosamine-6-sulfatase	GNS

Article title:

"Development of a 3D *in vitro* model of Dupuytren's Disease as a platform for drug screening"

Journal name:

Cellular and Molecular Bioengineering

Author names:

Jarmila Knitlova, Adam Eckhardt, Daniel Hadraba, David Vondrasek, Roman Stachon, Elena Filova, Vera Jencova, Kristyna Havlickova, Tatyana Kobets, Martin Ostadal and Lucie Bacakova

Affiliation:

Laboratory of Translational Metabolism,
Institute of Physiology of the Czech Academy of Sciences,
Videnska 1083, 142 00 Prague 4, Czech Republic;
+420 724 066 868

e-mail address of the corresponding author:

adam.eckhardt@fgu.cas.cz
