# Supplementary material for: Development of a 3D In Vitro Model of Dupuytren’s Disease as a Platform for Drug Screening
Source: Cell Mol Bioeng. 2026 Jan 19;19(1):111–27. doi: 10.1007/s12195-026-00885-2 (PMC13031596; doi:10.1007/s12195-026-00885-2)
Supplement: Supplementary file 6 — Analysis of polyacrylamide SDS gel electrophoresis [file 12195_2026_885_MOESM6_ESM.docx]

**Additional file 6**

**Title: Analysis of polyacrylamide SDS gel electrophoresis**

Individual collagen type I chains (α1, α2) were analyzed by 8% polyacrylamide SDS gel electrophoresis (the bands of collagen type I chains were confirmed by LC-MS). The stained gels were scanned with an imaging densitometer GS-800 (Bio-Rad), and protein bands were quantified by Quantity One software (Bio-Rad, v.4.6.8).


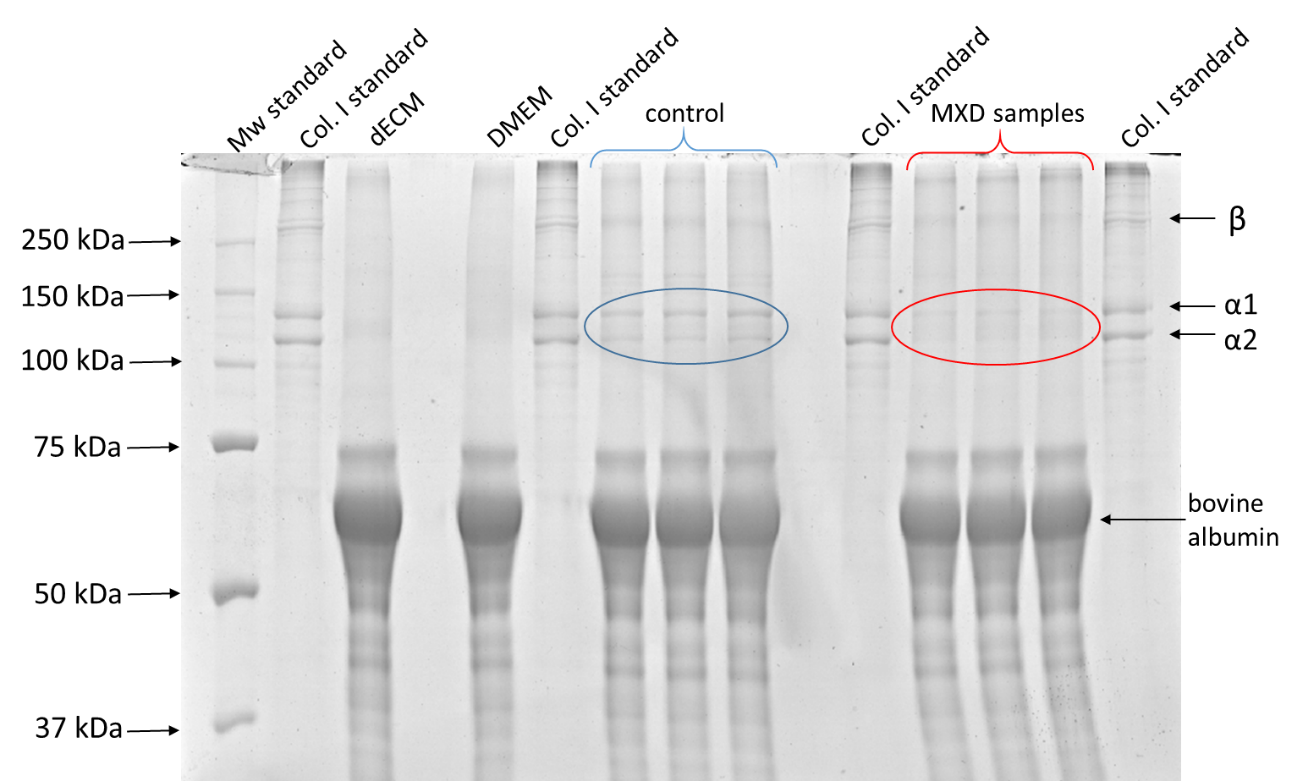


**Fig. S1**: Representative picture of a gel with collagen bands for densitometric analysis. The „dECM“ represents an acellular sample that was immersed in the culture medium in parallel with the recellularized samples. „DMEM“ sample represents DMEM cultivation medium supplemented with 2% fetal serum, 2.5ng/ml of TGF-β1 and 50 µg/ml of ascorbic acid. These samples were included as controls to ensure that we were not analyzing proteins originating from the culture medium (DMEM) or proteins released into the medium from the dECM during cultivation (dECM).

Article title:

“Development of a 3D *in vitro* model of Dupuytren’s Disease as a platform for drug screening”

Journal name:

Cellular and Molecular Bioengineering

Author names:

Jarmila Knitlova, Adam Eckhardt, Daniel Hadraba, David Vondrasek, Roman Stachon, Elena Filova, Vera Jencova, Kristyna Havlickova, Tatyana Kobets, Martin Ostadal and Lucie Bacakova

Corresponding author: Adam Eckhardt

Affiliation:

Laboratory of Translational Metabolism,

Institute of Physiology of the Czech Academy of Sciences,

Videnska 1083, 142 00 Prague 4, Czech Republic;

+420 724 066 868

e-mail address of the corresponding author:

[adam.eckhardt@fgu.cas.cz](mailto:adam.eckhardt@fgu.cas.cz)
